# The Cohesion Protein SOLO Associates with SMC1 and Is Required for Synapsis, Recombination, Homolog Bias and Cohesion and Pairing of Centromeres in Drosophila Meiosis

**DOI:** 10.1371/journal.pgen.1003637

**Published:** 2013-07-18

**Authors:** Rihui Yan, Bruce D. McKee

**Affiliations:** 1 Department of Biochemistry, Cellular and Molecular Biology, University of Tennessee, Knoxville, Tennessee, United States of America; 2 Genome Science and Technology Program, University of Tennessee, Knoxville, Tennessee, United States of America; Stowers Institute for Medical Research, United States of America

## Abstract

Cohesion between sister chromatids is mediated by cohesin and is essential for proper meiotic segregation of both sister chromatids and homologs. *solo* encodes a Drosophila meiosis-specific cohesion protein with no apparent sequence homology to cohesins that is required in male meiosis for centromere cohesion, proper orientation of sister centromeres and centromere enrichment of the cohesin subunit SMC1. In this study, we show that *solo* is involved in multiple aspects of meiosis in female Drosophila. Null mutations in *solo* caused the following phenotypes: 1) high frequencies of homolog and sister chromatid nondisjunction (NDJ) and sharply reduced frequencies of homolog exchange; 2) reduced transmission of a ring-X chromosome, an indicator of elevated frequencies of sister chromatid exchange (SCE); 3) premature loss of centromere pairing and cohesion during prophase I, as indicated by elevated foci counts of the centromere protein CID; 4) instability of the lateral elements (LE)s and central regions of synaptonemal complexes (SCs), as indicated by fragmented and spotty staining of the chromosome core/LE component SMC1 and the transverse filament protein C(3)G, respectively, at all stages of pachytene. SOLO and SMC1 are both enriched on centromeres throughout prophase I, co-align along the lateral elements of SCs and reciprocally co-immunoprecipitate from ovarian protein extracts. Our studies demonstrate that SOLO is closely associated with meiotic cohesin and required both for enrichment of cohesin on centromeres and stable assembly of cohesin into chromosome cores. These events underlie and are required for stable cohesion of centromeres, synapsis of homologous chromosomes, and a recombination mechanism that suppresses SCE to preferentially generate homolog crossovers (homolog bias). We propose that SOLO is a subunit of a specialized meiotic cohesin complex that mediates both centromeric and axial arm cohesion and promotes homolog bias as a component of chromosome cores.

## Author Summary

Sexual reproduction entails an intricate 2-step division called meiosis in which homologous chromosomes and sister chromatids are sequentially segregated to yield gametes (eggs and sperm) with exactly one copy of each chromosome. The Drosophila meiosis protein SOLO is essential for cohesion between sister chromatids. SOLO localizes to centromeres throughout meiosis where it collaborates with the conserved cohesin complex to enable sister centromeres to orient properly – to the same pole during the first division and to opposite poles during the second division. In *solo* mutants, sister chromatids become disconnected early in meiosis and segregate randomly through both meiotic divisions generating gametes with random (and mostly wrong) numbers of chromosomes. In this study we show that SOLO also localizes to chromosome arms where it is required to construct stable synaptonemal complexes that connect homologs while they recombine. In addition, SOLO is required to prevent crossovers between sister chromatids, as only homolog crossovers are useful for forming the interhomolog connections (chiasmata) needed for homolog segregation. SOLO collaborates with cohesin for these tasks as well. We propose that SOLO is a subunit of a specialized meiotic cohesin complex and a multi-purpose cohesion protein that regulates several meiotic processes needed for proper chromosome segregation.

## Introduction

Meiosis is a specialized type of cell division that generates haploid gametes from diploid germ cells. It encompasses a single round of DNA replication followed by two rounds of chromosome division in which first homologous chromosomes then sister chromatids segregate. During prophase of the first division (prophase I), homologous chromosomes pair, synapse and recombine with their partners. The resulting crossovers, stabilized by cohesion between sister chromatid arms, serve as chromatin linkers known as “chiasmata” that enable homolog pairs to bi-orient on the first division spindle. At anaphase I, resolution of sister chromatid arm cohesion leads to homolog segregation. Sister chromatids remain attached at their centromere regions until anaphase II, when resolution of centromere cohesion allows them to segregate [1]–[6].

Cohesion between sister chromatids is essential for several key steps in meiotic segregation and is mediated by ring-shaped cohesin complexes that embrace sister chromatid pairs [2], [7]. The subunits of cohesin are two SMC (structural maintenance of chromosomes) proteins, SMC1 and SMC3, and two non-SMC subunits, a “kleisin” subunit, which can be either the mitotic SCC1/RAD21 protein or its meiosis-specific paralog REC8, and a SCC3/SA-family subunit. SMC1 and SMC3 are long intramolecular coiled-coil proteins that form extended hairpin structures with N- and C-terminal globular ATPase domains at one end and a globular hinge domain at the other. SMC1 and SMC3 bind to each other at their hinge domains and to opposite ends of the kleisin subunit at their ATPase domains, forming a tripartite ring that embraces pairs of sister chromatids. The SA subunit binds to the kleisin subunit and regulates cohesin chromosome binding. Cohesin is loaded on chromatin prior to or during S phase and establishes cohesion during DNA replication. Although cohesin can be removed by other means and at other times in the cell cycle, cleavage of RAD21 or REC8 by the protease Separase at anaphase leads to release of sister chromatids and triggers segregation [7]–[10].

In meiosis, cohesion has a dual role, to keep homologs connected by stabilizing chiasmata on chromosome arms until anaphase I, and to keep sister chromatids connected at their centromere regions until anaphase II. The same cohesin complex, REC8 cohesin, is responsible for both arm and centromere cohesion and the same protease, Separase, is responsible for cleaving both arm cohesin at anaphase I and centromere cohesin at anaphase II. Since cohesin must be loaded prior to the first division, the centromeric cohesin complexes require protection from cleavage during anaphase I. This function is carried out by the centromeric guardian protein Shugoshin and its effectors (including the PP2A phosphatase) [11], [12]. REC8 and Shugoshin and the two-step cohesin release mechanism appear to be widely conserved [2], [7], [12]. REC8 and other cohesins are also required for several other essential steps during the first meiotic division, including homolog pairing, synapsis and recombination [2], [8], [9]. However, it is not clear to what degree these roles involve cohesion. In yeast and *C. elegans*, mutations in *rec8* and *smc3* can disrupt recombination, DSB formation and DSB repair without affecting cohesion [13], [14].

Another crucial meiosis-specific centromere modification, mono-orientation, is needed at the first division to prevent sister centromeres from connecting to opposite poles (bi-orienting) as they do at all other divisions. Instead, sister centromeres must collaborate in forming a single microtubule-binding surface and orient toward the same pole (mono-orient) so that their counterparts on the opposite homolog can orient to the opposite pole. This coordinated orientation of centromeres is essential to ensure that they segregate reductionally, with both sisters co-segregating to the same pole during the first meiotic division, rather than equationally as in mitosis or the second meiotic division. The mono-orientation process is not well understood. In *S. cerevisiae*, mono-orientation is mediated by a specialized Monopolin complex that clamps sister centromeres together, and a different specialized monopolin protein Moa1 is required for mono-orientation in *S. pombe*. However, these yeast proteins are not conserved. In several higher eukaryotes including *C. elegans* and *Arabidopsis*, cohesin is required for mono-orientation but what role it plays is not known [15]–[19].

Proper homolog segregation requires recombination to generate the crossovers that serve as chiasmata. Meiotic recombination is initiated by programmed double-strand breaks (DSBs) induced by the conserved Spo11 endonuclease [20]. Breaks are then repaired by a meiosis-specific version of the ubiquitous homologous recombination pathway modified to ensure that the repair products include adequate numbers of homolog crossovers (at least one per chromosome pair) [21], [22]. A crucial modification, known as “homolog bias”, involves preferential use of homologous over sister chromatids as repair templates, a reversal of the sister chromatid bias that prevails in somatic DSB repair [23], [24]. Understanding of the mechanism of homolog bias is rudimentary but studies in yeast have identified two groups of proteins that play key roles: the meiosis-specific recombinase DMC1, a paralog of RecA and RAD51, which preferentially mediates invasion of homologous rather than sister strands [25], [26]; and the SC proteins RED1, MEK1 and HOP1 that seem to function mainly by inhibiting sister chromatid exchange (SCE) [23], [24], [27]–[29]. The few proteins outside of yeast that have been identified as being important for homolog bias, including ORD in *Drosophila*, HIM-3 in *C. elegans*, and SYCP-2 and SYCP-3 in mammals are also SC proteins, pointing to a possible conserved function of the SC in homolog bias [11], [30]–[32].

Either before or coincident with the early stages of meiotic recombination (depending on organism), homologs pair and “synapse”, a process that culminates in assembly of a tripartite structure called synaptonemal complex (SC) [5]. SC consists of two parallel lateral elements (LEs) that encompass the axes of the homologs, connected by densely packed transverse filaments that span a central region of about 100 nm, and a central element that lies parallel to and midway between the LEs. Transverse filaments are homo-dimeric coiled-coil proteins that bind to each other at their N-termini and to the LEs at their C-termini [33], [34]. In many eukaryotes, the LEs are clearly visible prior to synapsis when they are called axial elements (AEs), but in Drosophila no AEs have been observed. Instead, the LEs and central regions of the SCs assemble simultaneously during synapsis [5], [6].

Synapsis initiates during zygotene as short stretches of SC assembled at axial association sites, accompanied or preceded (depending on species) by alignment of homologs [5], [6]. In some eukaryotes, axial association sites correspond to DSB sites where the early stages of interhomolog recombination take place [6]. However, in Drosophila, DSBs are delayed until pachytene when homologs are fully synapsed, and synapsis is initiated and completed independent of the recombination apparatus [35], [36]. The initial SC patches are extended by a poorly understood process that leads eventually, at pachytene, to fully aligned and synapsed homolog pairs. Recombination is thought to be completed during pachytene and after it is complete, the SCs are disassembled and homologs disassociate except at chiasmata, which keep them connected throughout the first division. [5], [6].

AE/LEs are prominent, meiosis-specific versions of chromosome axes that develop in early prophase I [5], [6]. They encompass the paired sister chromatid axes that anchor the chromatin loops and are built on a condensed “chromosome core” of densely packed cohesin complexes that serves as a scaffold for assembly of additional meiosis-specific AE/LE proteins that promote homolog interactions, mostly by mechanisms that remain to be defined [37], [38]. The best understood AE/LE proteins are RED1 and HOP1, mentioned above as yeast proteins involved in homolog bias. RED1 is also required for synapsis and SC formation but some other AE/LE proteins are dispensable for SC formation although they are often required to stabilize chromosome cores and SCs [27], [30]–[32], [39]–[43]. In mammals and Drosophila, homologous chromosome cores can synapse with each other in the absence of the non-cohesin AE/LE components although the resulting SCs tend to be unstable and to disassemble prematurely [37], [38], [43]. Many eukaryotes have additional meiosis-specific kleisin family members or other cohesin paralogs and many of these are found primarily or exclusively in cores [40], [44]. One such paralog is C(2)M, a kleisin family member in Drosophila that is present only during prophase I in cores and is required for LE assembly, synapsis and normal levels of recombination but is dispensable for cohesion [45], [46]. Thus current evidence points to a fundamental role of the cohesin-based chromosome cores in synapsis and SC structure. However, although cores are cohesin-based, the role of cohesion in chromosome core and SC assembly remains to be clarified.

Cohesion is essential for chromosome segregation in Drosophila meiosis as well, but the way in which cohesion is mediated appears to differ from most other eukaryotes. No true REC8 homolog has been identified. The aforementioned C(2)M is the only known meiosis-specific kleisin, but its role is much more specialized than REC8. It is an essential component of the chromosome cores and required for synapsis and recombination but it is not enriched at centromeres and has no apparent role in either arm or centromere cohesion [45], [46]. Orientation Disruptor (ORD) is a cohesion protein that seems to carry out many of the functions of REC8 but it is not, on the basis of primary sequence homology, a cohesin. ORD localizes to centromeres and is required for centromere cohesion in both male and female meiosis. ORD also localizes to LEs and although not required for assembly of LEs or SCs, it is required to prevent their premature fragmentation and dissolution. Finally, ORD is required for normal levels of homolog recombination and is the only Drosophila protein known to suppress SCE. Although not a cohesin by sequence homology, ORD localizes along with the SMC cohesin subunits both at centromeres and on LEs and likely carries out some or most of its functions in collaboration with cohesin. The case is particularly clear for centromere cohesion where *ord* mutations lead to depletion of centromeric SMC cohesins in both male and female meiosis [30], [43], [47]–[52].

We have recently described a second meiosis-specific Drosophila cohesion protein, SOLO [53]. SOLO is required for centromere cohesion in Drosophila male meiosis and its loss leads to failure of mono-orientation and random chromatid assortment. SOLO and SMC1 are both enriched near centromeres throughout meiosis until both proteins disappear at anaphase II. In a *mei-S332* (Shugoshin) mutant [11], both SMC1 and SOLO dissociate from centromeres simultaneously at anaphase I. In *solo* mutants, like *ord* mutants, centromeric SMC1 foci are absent at all stages of meiosis. Together these data indicate that SOLO functions in very close collaboration with the SMC1 cohesin subunit. However, like ORD, SOLO shows no sequence homology with cohesins, or with any other proteins in the database [53].

The previous study was limited to male meiosis in which homologs segregate by a unique mechanism that does not involve SCs, recombination or chiasmata. Instead a specialized conjunction complex holds homologs together in place of chiasmata [54]. SOLO is not required for any step in homolog segregation in males except for centromere mono-orientation [53]. In this paper we describe the roles of SOLO in Drosophila female meiosis and show that SOLO, like ORD, carries out a broad spectrum of meiotic functions that include cohesion, pairing and clustering of centromeres, regulation of chromatid orientation and segregation at both meiotic divisions, stable assembly of LEs and SCs, achievement of normal levels of homolog exchange, and suppression of sister chromatid exchange. We also show that SOLO and SMC1 reciprocally co-immunoprecipitate from ovarian protein extracts, further underlining the close cooperation between SOLO and cohesin. The very similar mutant phenotypes and lack of synergism between *solo* and *ord* mutations suggest that SOLO and ORD function together with cohesin in the same molecular processes. Overall, our data indicate that SOLO has essential roles in centromere cohesion, AE/LE stability and recombination. SOLO joins ORD as the second such protein to be identified in Drosophila. Analysis of the multiple functions of SOLO in meiosis should further insight into the roles of cohesion in meiotic segregation.

## Results

### *solo* mutations cause sister chromatid and homolog NDJ in female meiosis

Errors in meiotic chromosome segregation, referred to here as nondisjunction (NDJ), generate aneuploid gametes that can be detected and quantified in genetic crosses. X chromosome NDJ generates diplo-X and nullo-X eggs that yield distinctive progeny classes (matriclinous daughters and patriclinous sons) (Figure S1) in standard crosses. X NDJ frequencies were found to be highly elevated in females hemizygous for three different *solo* alleles, averaging 58.4% compared to 0% in the sibling wild-type (WT) control crosses (Table 1). Because the X chromosomes carried markers adjacent to and flanking the centromeres, the progeny that developed from diplo-X eggs could be analyzed for whether both X centromeres came from a pair of sister chromatids (referred to as sister chromatid (S) NDJ) or from homologous chromatids (referred to as homolog (H) NDJ). The relative frequencies of S and H NDJ were similar for the three alleles, averaging approximately 21% S NDJ. This figure may underestimate %S because of reduced viability of the homozygous S NDJ classes relative to the heterozygous H NDJ class.

**Table 1:** X chromosome nondisjunction in *solo* females.

	DJ^b^	DJ^b^	H^c^	S^d^	S^d^	nullo				
Genotypes^a^	B*♀*	B*^+^♂*	y^+^f^+^ *♀*	y^+^f*♀*	y f^+^♀	y B♂	N^e^	%NDJ^f^	%S^g^	P/F^h^
*solo^Z2-0198^*/*Df*	238	230	97	16	9	165	755	55.1	20.5	7.2
*solo^Z2-3534^*/*Df*	203	214	116	9	17	171	730	60.0	18.3	6.1
*solo^Z2-0338^*/*Df*	185	218	96	11	22	176	708	60.2	25.6	6.2
Total *solo*	626	662	309	36	48	512	2193	58.4	21.4	6.4
*solo/+*	1210	1031	0	0	0	0	2241	0.0		72.3

a *Dp(1;1)sc^v1^, y pn cv m f.y^+^/y* females of the indicated chromosome 2 genotypes were crossed with *YSX.YL, In(1)EN, y B/Y* males *(X∧Y/Y)*. *Df* = *Df(2L)A267* that is deficient for *solo*.

b DJ = normal (disjunctional) progeny.

c H = Matriclinous daughters derived from homolog NDJ.

d S = Matriclinous daughters derived from sister chromatid NDJ. See Figure S1 for classification of NDJ types.

e N = total number of progeny.

Since there was no significant difference among the three controls, the numbers were summed for the analysis.

f %NDJ = 100×2×NDJ flies/(N+NDJ flies) where NDJ flies = (H+S+nullo).

g %S = 100×S/(S+H).

h P/F = mean progeny per female.

NDJ of the autosomal 2^nd^ chromosome pair was also assayed (Table 2). Because of the inviability of 2^nd^ chromosome aneuploids, progeny derived from NDJ gametes are not recovered in crosses to chromosomally normal males. However, by crossing females to males carrying an attached-2 chromosome (*C(2)EN*), which generate only diplo-2 and nullo-2 sperm, NDJ eggs can be recovered when fertilized by reciprocally aneuploid sperm. This assay allows detection of NDJ but does not permit calculation of a NDJ frequency as no regular gametes are recovered. Crosses of *solo* females to *C(2)EN* males yielded 2.5 and 3.0 progeny/female for two different alleles, indicating the occurrence of chromosome 2 NDJ. Heterozygous *solo*/+ controls yielded no progeny in similar crosses. Since two maternal 2^nd^ chromosomes were recovered in half of the progeny, the relative frequencies of S and H NDJ could be measured. After correcting for viability differences, %S NDJ was estimated to be 32%, very near the expected frequency (33.3%) if chromatids segregate randomly at both meiotic divisions. These results indicate that *solo* causes NDJ of both sex chromosomes and autosomes and suggest that the NDJ mechanism might involve random chromatid assortment.

**Table 2:** Sister chromatid versus homolog NDJ for chromosome 2 in *solo* females.

Progeny Phenotypes	Egg genotypes	NDJ type	*solo^Z2-0198^*	*solo^Z2-3534^*
+	*b/cn bw*	Homolog	1012	259
Bw	*b bw/cn bw*	Homolog	36	10
b	*b/b*	Sister	144	38
cn bw	*cn bw/cn bw*	Sister	106	7
Cn	*cn/cn bw*	Sister	37	4
bw sp	nullo-2	Both	360	256

*solo, cn bw/b vas^7^* females were crossed singly to two *C(2)EN, bw sp* males. 2.48 and 3.02 progeny per female were recovered in the *Z2-0198* and *Z2-3534* crosses, respectively, indicating elevated NDJ frequencies. The phenotypes of the progeny, their presumed genotypes and origins and the numbers recovered are shown in the table. See Figure S2 for classifications of NDJ types. %S NDJ = 100×Sister/(Sister+Homolog). Correcting for viability effects, %S = 100× (144/0.5176+106/0.6349+37)/((144/0.5176+106/0.6349+37)+(1012+36)) = 32%. See Materials and Methods for methodology for viability correction. The viability of *solo^Z2-3534^ cn bw* homozygotes was not measured but they appeared to be poorly viable because they were rarely found in the *solo^Z2-3534^ cn bw/CyO* stock. Therefore the estimate of %S NDJ was based solely on the *solo^Z2-0198^* cross.

### *solo* mutations reduce homolog crossover frequencies

Crossover frequencies were measured in three euchromatic intervals, two (*pn-m* and *m-f*) that together encompass 80–85% of the recombinational length of the X chromosome and one (*cn-bw*) that encompasses about 90% of chromosome arm 2R, and in one mixed euchromatic/heterochromatic interval (*f-y^+^*) on the X chromosome (see Figures S1 and S2). For the X chromosome, exchange was measured in females hemizygous for each of the three *solo* alleles, using heterozygous (*solo*/+) siblings as controls to minimize background variation (Table 3). The chromosome 2 crosses were conducted similarly except that a null allele was used in place of the *Df* chromosome (Table 4). As the results for the three alleles did not differ significantly in either set of crosses for any of the intervals, combined results are also presented. Crossover frequencies decreased in all four intervals in the mutants, very substantially and uniformly (7.5- to 7.6-fold) in the three euchromatic intervals, and more moderately (26%) in the *f-y* ^+^ interval that encompasses the X centromere.

**Table 3:** X chromosome recombination in *solo* females.

Genotypes	*pn-m* b	*m-f* b	*f-y^+^* b	*pn-y^+^* b	progeny^c^
*solo^Z2-0198^*/*Df*	3.5	3	5.7	12.2	230
*solo^Z2-3534^*/*Df*	6.1	2.8	8.4	17.3	214
*solo^Z2-0338^*/*Df*	5	1.4	6.4	12.8	218
*solo* average	4.9 (13.1)	2.4 (13.2)	6.8 (73.9)	14.1 (21.8)	662
*solo/+* average	37.3	18.2	9.2	64.7	1031
*solo ord* a	6.6 (16.7)	2.4 (12.7)	2.4 (17.2)	11.4 (15.8)	425
*solo ord*/+	39.5	18.9	13.9	72.3	631

*Dp(1;1)sc^v1^, y pn cv m f.y^+^/y* females of the indicated chromosome 2 genotypes were crossed with *YSX.YL, In(1)EN, y B/Y* males *(X∧Y/Y)* and the B^+^ male progeny were scored to identify recombinants.

a *solo ord*: *solo^Z2-3534^ ord^Z2-5736^/solo^Z2-0198^ ord^5^*.

b map distance between different X markers in centiMorgans (cM); numbers in parentheses are the percentage of the control. *cv* was not scored in the test.

c Number of progeny scored in recombination analysis.

Since there were no significant differences among either the three *solo/+* controls or the three *solo/Df* experiments, combined results (*solo* average for the mutants and *solo*/+ average for the controls) are presented in addition to the results for the individual mutants. See Figure S1 for illustration and explanation of the cross.

**Table 4:** 2^nd^ chromosome recombination in *solo* and control females.

	Map Distance		
Female Genotypes^a^	cn bw^b^	N^c^	P/F^d^
*solo^Z2-0198^ cn bw*/*b vas^7^*	4.4 cM	317	6.8
*solo^Z2-3534^ cn bw*/*b vas^7^*	6.0 cM	251	5.3
*solo^Z2-0338^ cn bw*/*b vas^7^*	3.9 cM	282	5.0
*solo* average	4.77 cM (13.6)	850	5.7
*cn bw/b vas^7^*	35.9 cM	663	105.8
*b cn bw/+ + +*	41.9 cM	1167	77.8

a *solo, cn bw/b vas^7^* females were crossed singly with *b cn bw/CyO* males.

See Figure S2 for locations of 2^nd^ chromosome markers. The Cy^+^ progeny that inherited the *b cn bw* chromosome from the father were scored for the frequency of crossovers. Map distances are in cM.

b Numbers in parentheses represent the percentage of the sibling wild type control (*cn bw/b vas^7^*).

c Numbers of progeny scored in the recombination analysis.

d P/F = mean progeny per female.

The 7.6-fold reduction in crossovers between the distal (*pn*) and proximal (*f*) euchromatic X markers in our experiments falls within the fairly wide range of reported results for strong alleles of *ord* (6 to 20-fold reductions) and are in reasonable agreement with the reported 6.1-fold reduction for an *ord*-null genotype [47]–[49]. Based on very limited data, both *solo* and *ord* mutants cause similar reductions (6 to 10-fold) in frequencies of crossovers in euchromatic autosomal intervals as well (Table 4) [47], but have much weaker effects on exchange in intervals near or encompassing centromeres [47]–[49]. However, existing data do not reveal whether *ord* and *solo* function independently of each other in controlling exchange. To determine whether a *solo ord* double mutant would reduce exchange any further, we generated females that were trans-heterozygous for null alleles of both genes. Crossover frequencies in the X euchromatin (*pn-f* interval) were reduced 6.5-fold in the double mutants relative to *solo ord*/+ sibling controls (Table 3), a fold-reduction value intermediate between those of *ord* or *solo* single mutants. This result suggests that *solo* and *ord* function in the same recombination pathway, one that controls about 85–90% of crossovers along the X euchromatin and probably in autosomal euchromatin as well.

### *solo* is required for homolog bias

One way *solo* might function to promote homolog crossovers is by preventing recombination intermediates from being repaired by SCE. If so, *solo* mutations should increase SCE. Crossovers between sister chromatids cannot be detected in conventional recombination assays, but single (or other odd number of) crossovers between the chromatids of a circular (or “ring”) chromosome, generate double-ring dicentric chromosomes. In Drosophila females, the dicentrics generated by exchange between sister chromatids of a ring-X chromosome become trapped in unresolved bridges on the anaphase II spindle and are not transmitted. Since exchanges between sister chromatids of normal “rod” chromosomes have no consequence, the ratio of ring-X recovery to rod-X recovery among progeny of a ring-X/rod-X heterozygote is a rough measure of the SCE frequency. In previous studies, the ring-X/rod-X recovery ratio in WT control females ranged between 0.7 and 0.9 [30], [35], [45], [55], [56]. This likely reflects the normal background activity of the SCE pathway since in the absence of DSBs (i.e. in a *mei-P22* mutant), the ring-X chromosome is transmitted as efficiently as the rod-X [35], [57]. These results also show that the meiotic apparatus in Drosophila can transmit ring chromosomes efficiently as long as they are not dicentric. Several meiotic mutants have been analyzed by this assay but to date, mutations in only one gene, *ord*, have significantly reduced ring-X recovery [30], [35], [45], [55], [56].

To estimate meiotic SCE frequencies in *solo^Z2-0198^* and *solo^Z2-3534^* females, we measured the ring/rod recovery ratio in progeny of *solo/Df* or +/+ females heterozygous for the ring-X chromosome *Ring(1)2 (R(1)2)*. The ring/rod recovery ratios were 0.83 in the WT controls but only 0.35 and 0.36 in the *solo* crosses (Table 5). This result indicates that roughly 65 out of every 100 ring-X chromosomes were eliminated in *solo* meiosis. These results may actually underestimate the frequency of SCE because double ring-X crossovers, which might be quite frequent in *solo* mutants, yield normal mono-centric ring chromosomes which would not be detected in this assay. We conclude that *solo* mutations dramatically upregulate the SCE pathway, reversing the normal homolog bias to a sister bias.

**Table 5:** Sister chromatid exchange is increased in *solo* mutants.

Genotype	Ring progeny^a^	Rod progeny^a^	Ring/Rod
*R(1)2, y f/y w; +/+*	958	1156	0.83
*R(1)2, y f/y w; Df(2L)A267/solo^Z2-0198^*	209	605	0.35
*R(1)2, y f/y w; Df(2L)A267/solo^Z2-3534^*	216	604	0.36

The indicated females were crossed to *w^1118^/Y* males.

a Ring-X and Rod-X progeny were recognized by w^+^ (red) versus w (white) eyes, respectively.

See Materials and Methods for cross details. Only regular (disjunctional) progeny are included in the table and in the calculated ratios.

### *solo* mutations disrupt centromere pairing and cohesion

**Figure 1:**
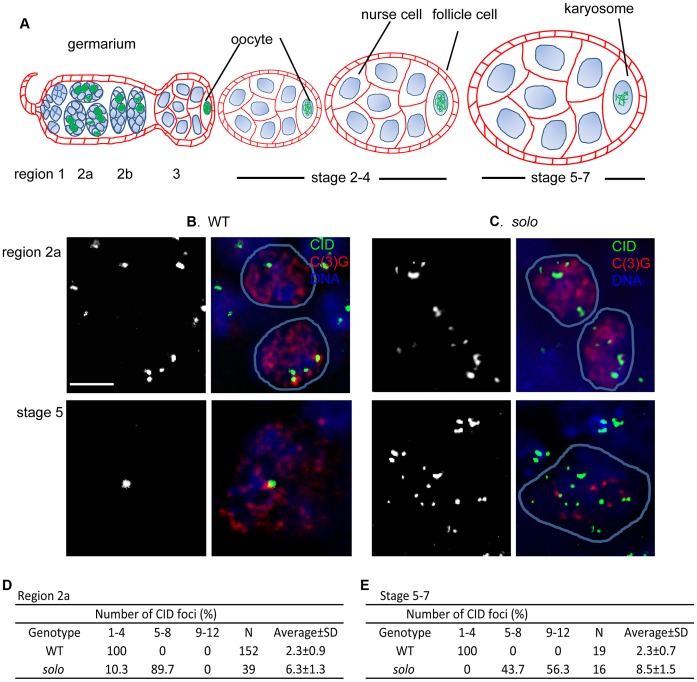
Centromere clustering, pairing and cohesion in *solo* and WT germ cells. (A) A schematic depiction of developmental stages of the germarium and early egg chambers of an ovariole [85], [86]. Stages are not drawn to scale. Meiosis is initiated within the germarium, the most anterior section of each ovariole. The germarium is divided into 4 regions (1, 2a, 2b and 3, anterior-posterior). Region1 contains germline stem cells (anterior tip), cystoblasts (posterior daughters of stem cells) and mitotically proliferating 2-, 4- and 8- cell cysts. 16-cell cysts initiate meiosis in region 2a with up to 4 cells/cyst assembling SCs in zygotene while the remaining cells (pro-nurse cells) begin differentiating into nurse cells. Only two cells (the pro-oocytes) continue into meiosis and assemble full-length SC in early pachytene (still region 2a). Cysts continue moving posteriorally and by region 3, full-length SC is restricted to a single oocyte, which lies at the posterior end of the cyst. As cysts continue to grow and mature, they leave the germarium and enter the vitellarium where they are encapsulated by a single layer of somatic follicle cells to form egg chambers. The chromosomes in the oocyte begin to condense around stage 3 and SC begins to disassemble around stage 4–5. By about stage 7, SC proteins can no longer be detected on chromosome arms and the chromosomes are condensed into a compact structure called the karyosome. (B, C) Centromeres in WT and *solo* germ cell nuclei. Centromeres, SC and DNA were stained by anti-CID antibody (green), anti-C(3)G antibody (red) and DAPI (blue), respectively. *solo*: *solo^Z2-3534^*/*Df(2L)A267*. WT: sibling control. Scale bar: 5 µm. (B) 1–4 CID foci were present in WT pro-oocyte and oocyte nuclei in early (top panel) and late (bottom panel) pachytene. (C) In *solo* mutants, 5–8 nuclear CID foci were present in most early pachytene nuclei (top panel) and more than 8 CID foci were present in most late pachytene nuclei (bottom panel). (D, E) Percentages of nuclei with indicated numbers of CID foci in pro-oocytes from region 2a (D) and in oocytes from stage 5–7 egg chambers (E). N = number of nuclei scored.

The recovery of both S and H NDJ progeny suggested that sister chromatid cohesion might be lost prior to the first meiotic division, as in *solo* males [53]. To test this idea, we used an antibody against Centromere IDentifier (CID), a centromere-specific histone H3 variant [58], [59] to examine centromere behavior during the first meiotic division in WT and *solo* ovaries (Figures 1B and 1C). The maximum number of CID spots during the first meiotic division would be 16 if all centromeres were separate. However, sister chromatid cohesion and homolog alignment, which are essentially complete in all WT pachytene nuclei, reduce the expected number of CID spots to a maximum of four. Moreover since non-homologous centromeres tend to cluster in prophase I, observed numbers are usually even fewer [43], [60], [61]. As expected, in WT ovarioles, C(3)G-positive nuclei from both region 2a germaria (early-mid-pachytene) and stage 5–7 egg chambers (late pachytene) exhibited 1–4 CID foci, averaging 2.3 at both stages (Figures 1B, 1D and 1E). In contrast, CID signals were much more numerous in *solo* pro-oocytes at all stages. In *solo* germaria only about 10% of pro-oocytes exhibited 4 or fewer spots, the remainder exhibiting 5–8 (mean = 6.3 (Figures 1C and 1D)). This suggests that both homologous centromere pairing and centromere clustering were disrupted by early-mid pachytene in *solo* mutants but that sister chromatid cohesion remained intact at this stage. However, by late pachytene (stage 5–7 egg chambers) more than half of the oocyte nuclei from *solo* ovaries exhibited more than 8 CID spots (Figures 1C and 1E), while the remainder exhibited 5–8 spots (mean = 8.5). Thus, in most oocyte nuclei, some sister centromere pairs had separated prematurely by the latter stages of pachytene. Very similar results were reported for an *ord* mutant [61]. Since prematurely separated sister centromeres are unlikely to establish mono-orientation on the spindle of the first meiotic division, these results may help explain the NDJ data.

### SOLO is expressed in oocytes and nurse cells and enriched at nuclear foci

To explore the expression pattern of SOLO in the female germline, we made use of two different transgenes expressing full-length SOLO cDNAs tagged with the enhanced yellow-fluorescent protein Venus. *UPS-SOLO::Venus* (*UPS-SOLO*) is driven by native regulatory sequences carried in a 2.7 Kb fragment of upstream genomic DNA. *UASp-Venus::SOLO* (*UAS-SOLO*) is controlled by GAL4-responsive UAS sequences [53]. Both transgenes were able to complement the NDJ phenotype of a null *solo* allele but the *UAS-SOLO* construct did so more robustly (Table S1). A single copy of the *UAS-SOLO* transgene, when expressed under control of the germline-specific driver *nos-GAL4::VP16* in a *solo* background, fully suppressed X chromosome NDJ. However, *solo* females carrying two to four copies of *UPS-SOLO* still underwent NDJ at modest but significant frequencies (7–11%). This difference cannot be explained by the location of the Venus tag because the C-terminally tagged SOLO protein completely rescued NDJ when expressed under control of *nos-GAL4::VP16* (Table S1) so may reflect a deficiency in expression level or pattern.

**Figure 2:**
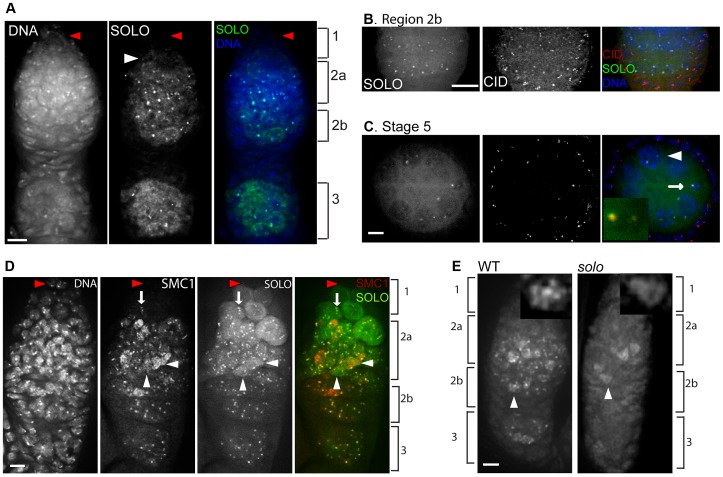
SOLO and SMC1 are enriched at centromeres and interact genetically. DNA was visualized with DAPI. Scale bars: 5 µm. (A–C) Localization of SOLO-Venus expressed from its native promoter. SOLO-Venus was detected with anti-GFP antibody. (A) SOLO-Venus formed bright foci and bound to chromosome arms in a whole-mount germarium. Females were deficient for the native *solo* gene but carried four copies of *UPS-SOLO::Venus* (*{UPS-SOLO::Venus}*; *Df(2L)A267/solo^Z2-0198^; {UPS-SOLO::Venus}*. SOLO foci were visible in mitotic cells in the posterior area of region 1 (white arrowhead) while no SOLO signals were seen in the anterior-most area of germarium that contains stem cells and early cystoblasts (red arrowheads). (B, C) *Df(2L)A267/solo^Z2-0198^; {UPS-SOLO::Venus}* females. SOLO formed bright foci at centromeres. CID was stained with anti-CID antibody. (B) SOLO and CID signals co-aligned in the nuclei of germ cells within region 2b but not in surrounding somatic follicle cells. (C) SOLO and CID signals co-aligned in the nuclei of germ cells but not somatic follicle cells in a stage 5 egg chamber. The oocyte (arrow) was identified by DNA amount (diploid) and position (posterior end of the cyst). The nurse cells are polyploid (arrowhead). The inset shows an enlarged view of the oocyte (arrow). (D) SOLO co-aligned with the cohesin component SMC1. Expression of Venus::SOLO was induced by *nos-Gal4::VP16* in *{UASp-Venus::SOLO}/{nos-Gal4::VP16}* females and stained with anti-GFP antibody. SMC1 was stained with anti-SMC1 antibody. No SOLO or SMC1 foci were found in the anterior-most tip of the germarium where stem cells and early cystoblasts reside (red arrowheads). SOLO and SMC1 localized to centromeres but did not form linear structures in the posterior of region 1 (arrows). SOLO and SMC1 formed linear structures on chromosome arms and bright foci at centromeres in region 2a (white arrowheads). (E) Dependence of SMC1 staining on SOLO. Bright SMC1 foci were completely absent in *solo* (*solo^Z2-3534^*/*Df*) germaria although some diffuse and linear SMC1 staining was still present. The insets are magnifications of pro-oocytes marked by the arrowheads and show linear staining in both WT and *solo* pro-oocytes.

In whole-mount ovarioles prepared from females lacking any functional copies of native *solo*, UPS-SOLO and UAS-SOLO exhibited overlapping but non-identical localization patterns (Figures 2A and 2D). Both proteins were expressed only in germ cells and in all regions of the germarium except for the anteriormost segment of region 1. The only really striking difference between the UPS-SOLO and UAS-SOLO expression patterns in whole-mount preparations was the considerably higher level of UAS-SOLO expression in a broad anterior domain that encompassed most of region 1 (except for the anterior tip) and anterior region 2a. As this domain coincides with the domain of highest expression of nos-GAL4, this is probably an ectopic over-expression effect.

In nearly all germ cells, both UAS-SOLO and UPS-SOLO exhibited small numbers of prominent bright nuclear foci and a broad diffuse pattern that appeared to encompass both cytoplasm and nucleus. In addition, some nuclei exhibited much fainter fibrillar or linear staining (discussed below). A distinctive aspect of the bright focal and diffuse staining patterns was the uniformity of expression level within cysts, indicating strong expression in both nurse and meiotic cells. Similar expression patterns were previously reported for ORD and the SMC cohesins [30], [43].

### SOLO is enriched at centromeres and is required for centromeric SMC1 foci

Bright foci of both UPS-SOLO and UAS-SOLO were observed in all germ cell nuclei in regions 2a, 2b and 3 of germaria and in egg chambers through at least stage 5. (UAS-SOLO signals have been detected as late as stage 8 (data not shown)). Fainter foci were also seen in some pre-meiotic nuclei in the posterior half of region 1. Most nuclei exhibited one to four SOLO foci per nucleus, suggesting that the foci may correspond to centromeres. This idea was tested by staining UPS-SOLO-expressing ovarioles with an antibody against CID. As shown in Figures 2B and 2C for germarial region 2b and a stage 5 egg chamber, all of the bright UPS-SOLO foci aligned with anti-CID signals, confirming that SOLO is enriched in the vicinity of centromeres in female germ cells. However, at higher magnification, the overlap between UPS-SOLO and CID foci sometimes appeared only partial (Figure 2C, inset) suggesting that SOLO may be enriched at pericentromeric domains as well as centromeric domains. UAS-SOLO foci aligned with anti-CID foci as well (data not shown).

SMC1 and SMC3 have been shown to be highly enriched on centromeres of female germ cells at similar stages [43]. To confirm co-enrichment of SOLO and SMC1 in females, we stained germaria expressing UAS-SOLO with an antibody against SMC1. As expected, the SMC1 signals formed bright nuclear foci throughout the germarium from posterior region 1 through region 3 in both meiotic cells and nurse cells (Figure 2D). As reported previously [43], and like SOLO, SMC1 signals were absent from the anterior tip of the germarium where germ line stem cells and cystoblasts reside. It is evident from Figure 2D that the bright SMC1 and UAS-SOLO foci overlap very extensively in germaria. They also overlap in later stages (data not shown). Thus, SOLO and SMC1 are co-enriched on meiotic centromeres in females as well as in males.

To test whether the centromeric SMC1 foci depend on *solo*, WT and *solo* germaria were stained with anti-SMC1 antibody. Whereas prominent SMC1 foci were present throughout the WT germarium, no SMC1 foci were detected in any nucleus in the *solo* germarium (Figure 2E). SMC1 foci were also absent from *solo* oocyte nuclei in later stages (data not shown). However, SMC1 staining did not disappear in *solo* germ cells. Diffuse staining was apparent in many germ cells in both WT and *solo* germaria, and appeared to be associated with chromosome arms (Figure 2E, arrowheads, insets). This staining pattern is explored further below. Thus, in female meiosis as in male meiosis, enrichment of the SMC1 subunit of cohesin at centromere regions is dependent on *solo*. However, SMC1 can localize to chromosome arms in the absence of *solo*.

### SOLO interacts with the cohesin subunit SMC1 *in vivo*

**Figure 3:**
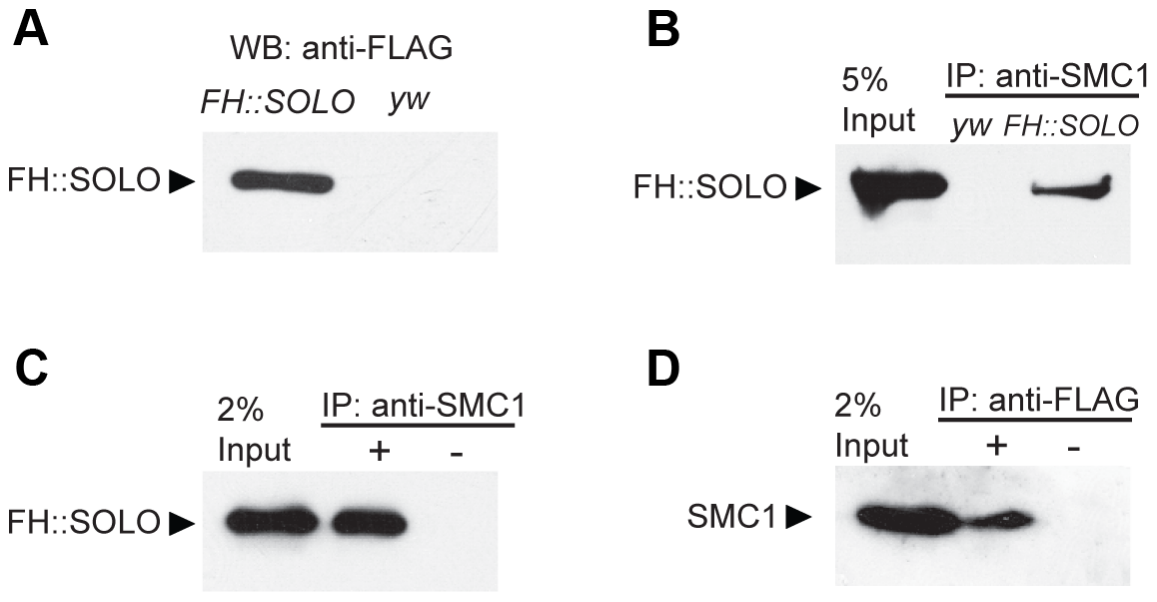
Co-immunoprecipitation of SOLO and SMC1 from ovarian extracts. Ovary lysates were prepared from *y w* and FH::SOLO (*{UASp-FH::SOLO}/CyO; {nos-GAL4::VP16}/TM2*) ovaries and used for Western blot and immunoprecipitation experiments. (A) Western blot of protein extracts from FH::SOLO and *y w* ovaries using anti-FLAG antibody. Absence of signal in the *y w* lane shows that the anti-FLAG antibody is specific. (B) Immunoprecipitation with anti-SMC1 antibody from protein extracts from FH::SOLO and *y w* ovaries, analyzed with anti-FLAG antibody. (C) Immunoprecipitation with anti-SMC1 antibody or rabbit serum (mimic control) from FH::SOLO ovary extracts, analyzed with anti-FLAG antibody. (D) Immunoprecipitation with anti-FLAG antibody or mouse serum (mimic control) from FH::SOLO ovary extracts, analyzed with anti-SMC1 antibody.

The findings that SOLO and SMC1 are co-enriched on centromeres and that SOLO is required for SMC1 localization to centromeres suggest that they may interact physically. In order to address this issue, we generated transgenic flies that express a full-length SOLO cDNA with tandem 3XFLAG and 3XHA tags at its N-terminus regulated by UAS sequences. One copy of this transgene completely reverted the NDJ phenotypes of *solo* males and females (Table S1) when induced by the germline-specific driver *nos*-*GAL4::VP16*, indicating that the FH::SOLO fusion protein is fully functional. Western blots revealed high level expression of FH::SOLO in ovaries. The absence of signal in the lane derived from *y w* (control stock lacking transgene) ovary extracts confirms the specificity of the anti-FLAG antibody (Figure 3A). In the co-immunoprecipitation experiment, FH::SOLO was pulled down from extracts of transgenic ovaries by the anti-SMC1 antibody (Figure 3B) used in immunofluorescence experiments in this and previous studies [53], [54] but not by host control serum (Figure 3C). To rule out the possibility that FH::SOLO could be precipitated by cross-reactivity from the anti-SMC1 antibody, the reciprocal immunoprecipitation, i.e., using anti-FLAG antibody to immunoprecipitate SMC1, was carried out and the result showed that SMC1 was co-immunoprecipitated by anti-FLAG antibody (Figure 3D). Our results demonstrate that SOLO associates *in vivo* with SMC1, one of the core components of the cohesin complex.

### SOLO signals align with SMC1 and C(3)G signals on synaptonemal complexes

**Figure 4:**
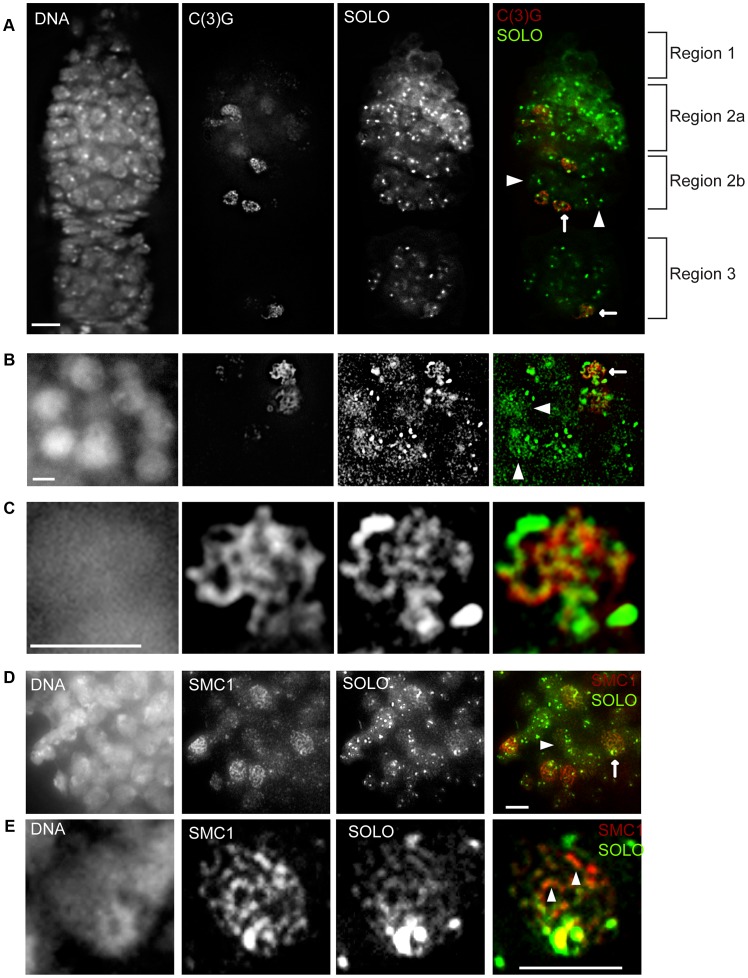
SOLO co-aligns with C(3)G and SMC1 on chromosome arms in pro-oocytes and oocytes. Expression of Venus::SOLO was induced by *nos-GAL4::VP16* in *Df(2L)A267/solo^Z2-0198^; {UASp-Venus::SOLO}/{nos-GAL4::VP16}* females and stained with anti-GFP antibody. SC was visualized by anti-C(3)G staining and DNA was stained with DAPI. Scale bars: 5 µm. (A) SOLO and C(3)G staining in a whole-mount germarium. Venus::SOLO localized to pro-nurse cells (no C(3)G staining, arrowheads) and to pro-oocytes. Faint linear Venus::SOLO signals could be discerned in some pro-oocytes and oocytes (arrows). (B–C) Venus::SOLO localization in germ cell nuclei prepared by chromosome spread method. Linear Venus::SOLO signals aligned with linear C(3)G staining (arrow) in pro-oocytes. Venus::SOLO also localized as bright foci and thin lines in pro-nurse cells (arrowheads). (C) Magnification of a pro-oocyte marked by the arrow in (B). (D and E) Co-alignment of SOLO and SMC1 signals in germ cell nuclei. Chromosomes were prepared by chromosome spreading. Pro-oocyte (arrow) and pro-nurse cell (arrowhead) are marked. (E) Magnification of a pro-oocyte marked by the arrow in (D). Arrowheads point to SC segments with well-aligned SOLO and SMC1 signals in which SMC1 signal is significantly brighter than SOLO signal.

The whole-mount preparations of germaria in Figures 2D, 2E and 4A show prominent linear signals of SMC1 and C(3)G in a subset of germ cell nuclei throughout regions 2–3. Based on previous studies, these structures are presumed to correspond to the LEs and central regions, respectively, of SCs [30], [33], [43], [45], [60]–[63]. Although it was less obvious in whole mount preparations, SOLO also localized to linear structures in pro-oocytes and oocytes (Figure 2D, arrowheads, and Figure 4A, arrows). To permit detailed comparisons of these patterns, chromosome spread preparations from UAS-SOLO germaria were stained with antibodies against C(3)G or SMC1. Linear UAS-SOLO signals, presumed to represent staining of chromosome arms, could be clearly seen in meiotic cells (Figures 4B and 4D, arrows), as identified by C(3)G or SMC1 linear structures, but were not confined to the meiotic cells. Thinner linear signals could be discerned in many pro-nurse cells in the same cysts (arrowheads). The same was true for SMC1 (Figure 4D, arrowhead), as previously reported [43], but not for C(3)G (Figure 4B, arrowheads), which is expressed in a meiosis-specific pattern. The thin linear UAS-SOLO and SMC1 signals in pro-nurse cells (Figure 4D, arrowhead) appeared to co-align extensively, similar to ORD and SMC1 [43].

Detailed comparisons of the ribbon-like localization patterns of UAS-SOLO with those of C(3)G and SMC1 in pro-oocytes were possible from magnified images such as those in Figures 4C and 4E. It is apparent from these images that the ribbon-like UAS-SOLO signals overlap quite extensively with the corresponding structures of SMC1 and C(3)G. The overlap is nearly complete for UAS-SOLO and SMC1. Although there were a few prominent segments that exhibited stronger SMC1 signals than UAS-SOLO signals (Figure 4E, arrowheads) and other segments with the reverse pattern, there were no segments of significant length that stained with SMC1 but not UAS-SOLO or vice versa. The overlap between UAS-SOLO and C(3)G was also very substantial but with more segments in which staining was quite unequal (Figure 4C). These results suggest that SOLO is widely distributed along SCs during pachytene and closely aligned with the cohesin SMC1, a pattern consistent with a possible role of SOLO as a component of Drosophila LEs.

To be sure that the results with the ectopically-driven UAS-SOLO were physiologically meaningful, we also carried out chromosome spread experiments using UPS-SOLO germ cells stained with anti-C(3)G (Figure S3). Like UAS-SOLO, UPS-SOLO localized to chromosome arms in pro-nurse cells (lower panels) and along C(3)G ribbon-like structures in pro-oocytes and oocytes (upper panels). However, UPS-SOLO signals were weaker than UAS-SOLO signals, and staining of the LEs was patchy and discontinuous rather than continuous. It is unclear at this point which pattern is correct. The fact that UPS-SOLO failed to fully rescue the X NDJ phenotype may indicate that its expression level is lower than the native gene. However, we cannot rule out the possibility that the more continuous SC labeling pattern of UAS-SOLO is due to overexpression and is therefore misleading. A transgene that expresses SOLO at native levels and fully rescues *solo* mutants will be required to resolve this question. Overall, these data indicate that SOLO localizes along chromosome arms in a pattern largely parallel to that of SC proteins, suggesting it may have a role in SC formation.

### Synaptonemal complex morphology is abnormal in *solo* females

To assess the effects of *solo* mutations on SC formation, we stained dissected ovaries with antibodies against C(3)G and ORB. ORB is a cytoplasmic protein that is present in all cells in most pachytene cysts, but substantially enriched in pro-oocytes and oocytes [64]. Synapsis phenotypes were analyzed for two different *solo* alleles (*solo^Z2-0198^* and *solo^Z2-3534^*), both of which are genetic null alleles for the NDJ phenotypes [53] (Table 1 and unpublished data).

**Figure 5:**
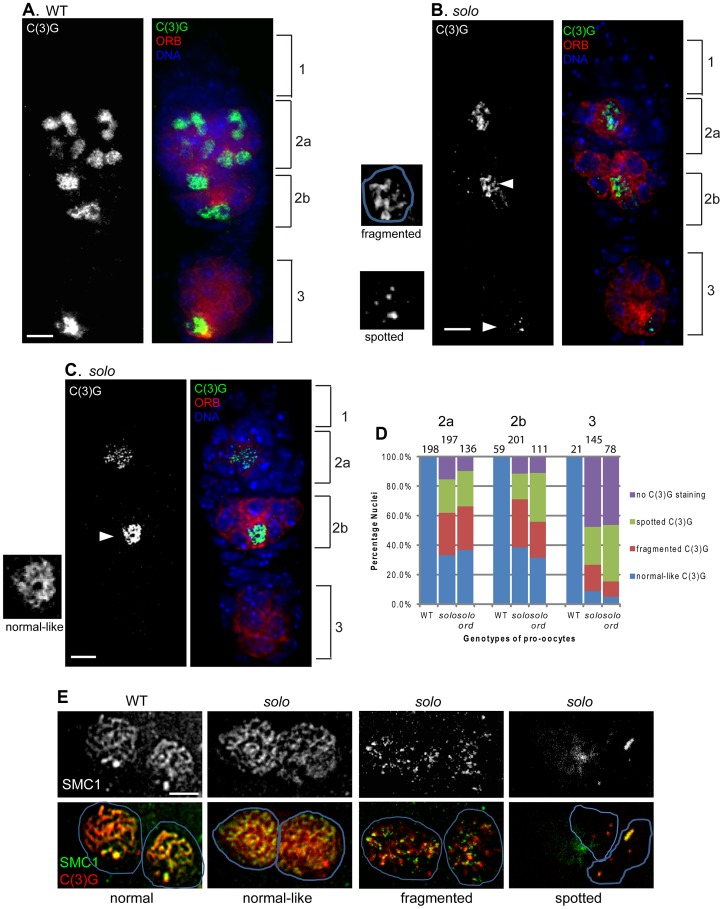
*solo* mutations destabilize lateral elements and central regions of SCs. Scale bar: 5 µm. (A–D) *solo* mutations caused defective C(3)G staining. Pro-oocytes and oocytes were stained by anti-ORB antibody. SC was visualized by anti-C(3)G staining and DNA was stained with DAPI. (A) In WT germaria C(3)G formed linear structures in nuclei of ORB-enriched cells within region 2a, 2b and 3, and was restricted to the oocyte in region 3. (B, C) In *solo* (*solo^Z2-3534^*/*Df(2L)A267*) germaria, pro-oocytes and oocytes were marked by enriched ORB staining. C(3)G staining patterns included spotted and fragmented (B) and normal-like (C) and are displayed in magnifications of cells marked with arrowheads. Note also the absence of C(3)G staining in the cyst in region 3. (Note: nuclei that appeared fully stained with C(3)G and did not exhibit obvious fragmentation were classified as “normal-like” even if the staining pattern did not look completely normal.) (D) Quantification of C(3)G defects in *solo* and *solo ord* pro-oocytes and oocytes. The graph shows the percentages of nuclei from ORB-enriched cells with normal-like, fragmented, spotted and no C(3)G staining. The numbers of pro-oocytes or oocytes scored are noted for each bar. (E) Abnormal SMC1 and C(3)G linear structures in *solo* pro-oocytes prepared by chromosome spreading and stained with both anti-SMC1 and anti-C(3)G antibodies. SMC1 exhibited normal-like, fragmented and spotted staining patterns that closely paralleled the patterns of C(3)G staining.

In *solo* mutant germaria, both ORB-staining and C(3)G staining were significantly reduced relative to WT germaria. The reduction in staining resulted from two distinct phenotypes: first, a substantial reduction in the numbers of germ-cell cysts per germarium; and second, reduced and/or morphologically abnormal C(3)G staining in many pro-oocytes and oocytes (Figures 5 and S4). However, no defect in oocyte specification was observed. Cysts in region 3 and later stages nearly always had only one cell with enriched ORB staining and no more than one cell with C(3)G staining, (e.g., Figures 5B, 5C, S5B and S6B) although C(3)G staining could be completely absent (e.g., Figure 5C), as described below.

Further analysis revealed that the first phenotype is due not to loss of *solo* function but instead to an unexpected and, as yet, unexplained inhibitory effect of the *solo* alleles on expression of *vasa*, a gene with an overlapping transcription unit that is required for early germ-cell development [53], [65]. Expression of a GFP-VAS transgene in *solo/Df* females substantially improved the germ-cell cyst number phenotype (Figure S5) and nearly doubled fertility (Table S2) but did not improve either the abnormal C(3)G staining patterns (second phenotype) (Figure S6) or the fidelity of chromosome segregation (Table S2). This shows that the abnormal C(3)G staining patterns are due to loss of *solo* function, not to reduced *vasa* function, and will be our focus in the following sections.

The C(3)G staining defects caused by the *solo* mutations were observed in cells with enriched ORB staining, marking them as pro-oocytes or oocytes, and fell into three main phenotypic categories: i) cells with partial or fragmentary staining; ii) cells with no linear segments at all but only C(3)G foci (spotty staining); and iii) cells that should have exhibited C(3)G staining based on ORB-staining but did not (no staining) (Figures 5B, 5C and S6B). A fourth category consisted of cells with nuclei that appeared to be fully stained and did not exhibit any obvious fragmentation; these were referred to as “normal-like” even though the staining patterns in these cells were often less clearly defined than in WT. Quantitative analysis showed that the three abnormal patterns, fragmentary, spotty, and no staining, were present at highly elevated frequencies, compared to WT, at all pachytene stages in *solo* germaria (Figure 5D). The quantitative analysis also revealed a progressive deterioration in C(3)G staining with increasing age of cyst. 30–40% of ORB-enriched cells in regions 2a or 2b exhibited normal-like C(3)G staining but that frequency declined to less than 10% by region 3. Some C(3)G staining persisted in some late pachytene oocytes (e.g., Figure S4), but many lacked staining altogether. Staining defects were not limited to C(3)G. SMC1 staining patterns exhibited a similar spectrum of defects (Figure 5E) with very similar frequencies of staining categories (data not shown). Moreover, when the C(3)G and SMC1 staining patterns were compared in the same cells by dual immunostaining, the patterns were very similar, as illustrated by the *solo* panel series in Figure 5E. Overall, these data indicate that *solo* mutants cause fragmentation and degeneration of LEs and SCs from the onset of pachytene and that these phenotypes worsen as cysts age.

### Do *solo* and *ord* interact?

The phenotypes caused by mutations in *solo* and *ord* are very similar in most respects, including the progressive fragmentation and disintegration of both SCs and chromosome cores during pachytene [30], [43]. However, there is a significant difference in the time of onset of abnormalities between *solo* and *ord* mutants. Whereas the phenotype is already present at high frequency in region 2a in *solo* mutants, it doesn't manifest to a significant degree until late stage 2a/stage 2b in *ord* mutants. To better understand the relationship between these phenotypes, we constructed *solo ord* double mutants and compared the C(3)G staining patterns to those in *ord* and *solo* single mutants. Whereas *ord* germaria exhibited normal C(3)G staining in region 2a, abnormal C(3)G staining patterns were seen in *solo ord* germaria at all stages (Figure S7) and did not differ significantly from the pattern in *solo* mutants (Figure 5D). Why *solo* mutants disrupt synapsis earlier in pachytene than *ord* mutants remains to be determined.

### *solo* mutants cause a transient delay in repair of meiotic DSBs

**Figure 6:**
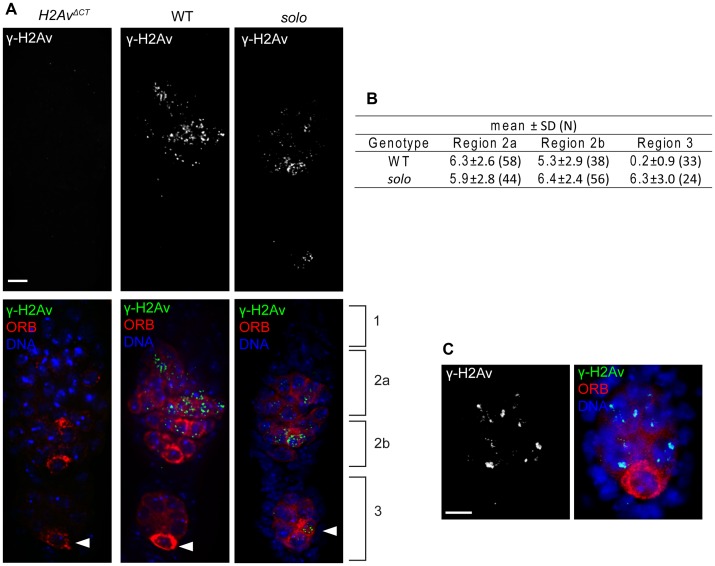
*solo* mutations cause a transient delay in DSB repair. Pro-oocytes and oocytes from *solo* mutant (*solo^Z2-3534^*/*Df(2L)A267*) and WT females were identified and staged by ORB staining and relative positions within germarium. γ-H2Av was stained by anti- γ-H2Av antibody and DNA was visualized with DAPI. γ-H2Av foci from pro-nurse cells were not scored. Scale bars: 5 µm. (A) γ-H2Av staining in germaria. Left panel shows absence of antibody staining in *P{w+H2Av^ΔCTXC^}; l(3)H2Av^810^* females in which the only expressed histone H2Av subunits are deficient for serine-137 and are not phosphorylated in response to DSBs [66]. Center and right panels show antibody staining in WT and *solo* germaria. γ-H2Av staining is absent in region 3 oocyte in WT but present in region 3 oocyte in *solo* (arrowheads). (B) Average number of γ-H2Av foci per nucleus in pro-oocytes and oocytes at different stages. N is the number of nuclei scored. (C) Absence of γ-H2Av staining in the oocyte nucleus of a stage 2 *solo* egg chamber. Egg chamber was from the same *solo* ovariole from which the germarium shown in panel A was taken.

The effect of *solo* on homolog exchange could reflect a defect either in formation or repair of meiotic DSBs. To address these possibilities, DSB frequencies were estimated in pro-oocyte and oocyte nuclei in *solo* and WT germaria using an antibody against γ-H2Av, a phosphorylated form of the histone variant H2Av protein that becomes enriched around DSBs shortly after their formation and that disappears when DSBs are repaired [66], [67]. γ-H2Av foci and/or short stretches were absent in region 1 germ cells from both *solo* and WT germaria but were present in pro-oocyte nuclei in regions 2a and 2b in both genotypes, consistent with previous reports [36], [68]. Although *solo* germaria exhibited fewer total foci than WT germaria, the two genotypes did not differ significantly in mean number of foci per pro-oocyte nucleus, indicating that the DSB formation is not impaired in *solo* mutants (Figures 6A and 6B). By contrast, unlike in WT, γ-H2Av foci were not restricted to region 2 in *solo* germaria. All 24 ORB-stained region 3 oocytes that were scored in *solo* germaria exhibited foci. The mean focus numbers did not differ significantly between region 3 and region 2 (Figures 6A, 6B and S8), suggesting a delay in DNA repair. However, foci did not persist beyond region 3; nearly all stage 2 oocytes and all stage 3 oocytes in *solo* mutants lacked γ-H2Av signals (Figure 6C). In this regard, *solo* mutants differ from DSB repair pathway mutants such as *spnA, spnB* and *spnD*, in which γ-H2Av foci persist until late pachytene [36], [68]–[71].

In principle, the delayed disappearance of γ-H2Av foci in *solo* mutants could reflect delayed germ cell development due to the effect of *solo* mutations on *vasa* function. In other words, if most region 3 oocytes in *solo* germaria are really at a developmental age typical of region 2a or 2b pro-oocytes in WT, then the persistence of foci in region 3 would have a trivial explanation. If this were the case, one would expect to see other evidence of delayed development such as failure to restrict ORB staining to a single cell. However, as described above, this was not the case. Nevertheless, to be sure that reduced *vasa* expression was not somehow responsible for the delayed disappearance of γ-H2Av foci, we compared the γ-H2Av phenotypes of *solo*; *GFP::VAS* and *solo* females. Similar to *solo* mutants, γ-H2Av foci persisted in region 3 oocytes but were absent in stage 2 oocytes of *solo/Df; GFP::VAS/+* (Figure S9) and *solo/Df; GFP::VAS/GFP::VAS* (data not shown). Thus the delayed disappearance of γ-H2Av foci exhibited by *solo* mutant females is not due to the effect of the *solo* mutation on *vasa* function. These results indicate that *solo* mutations have no effect on DSB formation but cause a transient delay in DSB repair. The cause of this delay and its significance with respect to the recombination phenotype of *solo* mutants are unknown.

## Discussion

### SOLO is required for multiple steps in the meiotic segregation pathway

Our previous analysis of *solo* in Drosophila male meiosis showed it to be essential for meiotic centromere cohesion and centromere orientation. However, the idiosyncratic homolog segregation mechanism in males precluded analysis of roles of *solo* in homolog interactions [53], [54]. In this study we analyzed the role of *solo* in female meiosis and found that *solo* mutations disrupt a much broader range of meiotic processes in females, including centromere clustering, homologous centromere pairing, sister centromere cohesion, sister centromere mono-orientation, SC and lateral element stability, homolog exchange, and homolog bias. Moreover, SOLO protein localized to chromosome arms and along the LEs of the SCs as well as to centromeres in female meiosis. These results indicate that SOLO contributes to multiple sister chromatid and homolog interactions that underlie meiotic chromosome segregation.

### NDJ and centromere cohesion in *solo* mutants

*solo* mutations severely disrupted chromosome segregation, causing X chromosome NDJ at frequencies in excess of 50% (Table 1). The NDJ pattern, a 1∶2 ratio of sister chromatid to homolog NDJ seen also in male *solo* mutants and *ord* mutants of both sexes, is consistent with random chromatid assortment caused by loss of centromere cohesion prior to prometaphase I [48], [50], [53]. Centromere cohesion was visibly impaired by late pachytene in *solo* females, based on CID spot numbers that consistently exceeded eight per cell (Figure 1). Similar observations were reported for *ord* mutants in female and male meiosis [52], [61] and *solo* mutants in male meiosis [53]. Although cytological analysis of segregation in *solo* females has not been undertaken, FISH analysis in *solo* males revealed random co-segregation of chromatids at anaphase I, fully separated chromatids by mid-anaphase I and chaotic segregation at anaphase II [53] and several cytological studies of segregation in *ord* males and females have documented premature sister chromatid separation and disorderly segregation behavior [47]–[52], [72].

The mechanism by which *solo* controls centromere cohesion seems likely to involve cohesin. In male meiosis, SOLO, ORD and SMC1 are enriched on centromeres until anaphase II and all three proteins depend on the Shugoshin ortholog MEI-S332 for maintenance on centromeres after metaphase I [43], [52], [53]. In female meiosis, SOLO, ORD, SMC1 and SMC3 are all enriched on centromeres in female meiosis throughout pachytene (Figures 2 and 4) [30], [43]. When either *solo* or *ord* is mutated, no centromeric SMC cohesin foci have been detected at any stage in either sex (Figure 2) [43], [53] with the consequences summarized above. These data are consistent with the hypothesis that centromere cohesion is mediated in male and female meiosis by centromere enrichment of a cohesin complex dependent on both SOLO and ORD. However, there has been no direct demonstration that the cohesive roles of SOLO and ORD are limited entirely to regulating cohesin. There also remains no direct evidence that any of these proteins – SMC1, SMC3, ORD or SOLO – persists on centromeres after pachytene. That may be a technical detection issue of some sort but until such evidence is obtained, the possibility that cohesion is maintained during the division stages in female meiosis by some other complex cannot be ruled out.

It is worth noting that centromere cohesion persisted intact throughout early and mid-pachytene *in solo* mutants despite the absence of detectable SMC1 centromere foci at any stage (Figure 1). Similar observations have been reported for *solo* male meiosis and *ord* male and female meiosis [51]–[53], [60], [61], [73] and indicate the existence of centromere cohesion that is independent of both SOLO and ORD and perhaps of cohesin (although the possible presence of low levels of cohesin near centromeres in *solo* and *ord* mutants cannot be ruled out). Whether this early prophase cohesion is based on a protein complex or on chromatid entanglement remains to be determined.

### Centromere pairing and clustering

Homologous centromeres are paired in nearly all germ cells and they further coalesce into 1–3 clusters at the onset of meiosis in pro-oocytes and remain paired and clustered throughout prophase I [43], [60], [61], [74]. There is considerable evidence that centromeric or heterochromatic associations between homologs underlie the robust achiasmate segregation system in Drosophila [74]–[77]. Moreover, centromere clusters serve as the first synapsis initiation sites during zygotene, accumulating the transverse filament protein C(3)G and the central element protein CONA [34], [60], [61], [78]. Both pairing and clustering (as well as synapsis initiation) was shown to depend on *ord* [43], [60], [61]. Here we demonstrate that *solo* is also required for these events. In early-mid pachytene *solo* pro-oocytes exhibited 6.3 foci per nucleus compared to 2.3 in WT, indicating substantial loss of pairing and clustering (Figure 1). Since SOLO and ORD are required for centromere enrichment of SMC1 as well as for centromere pairing and clustering, a logical inference is that centromere pairing is also mediated by cohesin, as previously suggested [60]. This suggestion is supported by evidence that centromere pairing is weakened in certain chromosomal backgrounds by reducing SMC1 gene copy number [79]. The mechanism by which cohesin mediates pairing and clustering is not known. Clustering may involve recruitment of SC proteins since mutations in *c(3)G* and *cona* abolished clustering [61]. However, *c(3)G* and *cona* mutations had much weaker effects on centromere pairing suggesting other mechanisms are probably involved in this process. Interestingly, yeast REC8 is also required for centromere pairing (called coupling) in early prophase I and promotes pairing by recruiting the yeast version of C(3)G, ZIP1 [80]. However, the relevance is not clear since centromere coupling in yeast is entirely promiscuous whereas Drosophila pairing is homologous [51]–[53], [73]–[76]. The mechanistic relationship between cohesion and centromere pairing remains to be elucidated. Given the association between centromere pairing and synapsis, it will also be of interest to investigate the role of *solo* in synapsis initiation.

### SOLO, ORD and cohesin

What are the roles of ORD and SOLO in cohesin function? Neither protein exhibits significant homology to any of the four cohesin protein families [49], [53], yet they appear to co-localize with SMC cohesins and are required for enrichment of SMC cohesins at centromeres. We favor the idea that SOLO and ORD are subunits of a meiosis-specific cohesion complex that includes the SMC subunits. ORD and SOLO may function to replace the canonical non-SMC subunits which, with the exception of C(2)M, have yet to be identified in Drosophila meiosis. Our finding that SOLO and SMC1 reciprocally co-immunoprecipitate from ovarian protein extracts is consistent with this idea but also with alternatives such as that SOLO is a regulator rather than a subunit of cohesin. More detailed biochemical analyses will be required to resolve the composition of Drosophila meiotic cohesin and to clarify the roles of SOLO and ORD.

### SOLO and arm cohesion

Cohesion between sister chromatid axes is clearly essential for maintenance of chiasmata but its role in early prophase I events such as homolog pairing, synapsis and meiotic recombination is unclear. In WT Drosophila, FISH studies indicate that sister chromatid arm sequences are tightly cohesive throughout prophase I [30], [74], but the genetic basis for arm cohesion remains to be elucidated. In *c(2)M* mutants, recombinant chromatids were not recovered in NDJ gametes, suggesting that chiasmata are stable and can bi-orient bivalents [45]. In *ord* mutants, absence of metaphase I arrest indicated an absence of chiasmata [51]. Presumably this implies that ORD also provides arm cohesion during prophase I and C(2)M does not, but direct evidence is lacking. ORD and the SMC cohesins are abundant on chromosome arms in all cells in 16-cell germ-line cysts, but *ord* mutants have little if any effect on intensity of SMC1/3 arm staining even in pro-oocytes and oocytes with fragmented cores [43]. Moreover, the limited FISH analysis that has been carried out thus far has not detected any disruption of arm cohesion during prophase I in *ord* mutants [30].

Our data show that SOLO is also expressed in all cells in 16-cell germline cysts and localizes to chromosome arms in both pro-nurse cells and pro-oocytes and oocytes. UAS-SOLO and UPS-SOLO are fully consistent in this respect (Figures 2, 4 and S3). In spread preparations co-stained with anti-SMC1 it is quite clear that the two proteins co-align very strongly even though the staining lines are thin (Figure 4D). These data suggest that SOLO may also be involved in arm cohesion. This is an important question for future research because the roles of *solo* in synapsis, chromosome core stability and recombination could be related to its role in arm cohesion.

### Role of SOLO in chromosome cores

Our data show that SOLO localizes to extended ribbon-like structures on the chromosome arms of pro-oocytes and oocytes, where it co-aligns with both SMC1 and C(3)G. This localization pattern is unlikely to be an artifact since it was seen with both UAS-SOLO and UPS-SOLO. However, it remains unclear whether the true pattern is the continuous staining pattern seen with UAS-SOLO or the discontinuous pattern seen with UPS-SOLO. Since UAS-SOLO appeared to be somewhat overexpressed in anterior 2a, the continuous localization could be an overexpression artifact. However, since UPS-SOLO did not fully rescue X chromosome NDJ in *solo* females, the discontinuous localization pattern could be an underexpression artifact. For now, we favor the continuous pattern in part because ORD localizes continuously [30], [43] and the phenotypes of *ord* and *solo* are so similar that sharply different localization patterns seem unlikely. Ascertaining the true localization pattern is an important goal.

Where exactly does SOLO localize? Overall, SOLO appeared to align slightly better with SMC1 than with C(3)G. However, this difference is not large and would not in itself suffice to assign SOLO to the LEs rather than the central regions. There are two independent reasons to favor the LEs. First, the close alignment of SOLO and SMC1 signals along unsynapsed chromosome arms of germ cells would likely persist during core assembly (Figure 4). Second, the highly correlated SMC1 and C(3)G staining phenotypes in *solo* mutants suggest that *solo* controls chromosome core stability directly rather than indirectly through effects on the central region, as even null mutations in *c(3)G* do not perturb chromosome core integrity (Figure 5) [45]. In other eukaryotes a distinction is often made between chromosome core proteins, which are cohesins, and non-core AE/LE proteins such as RED1, HOP1, SYCP-2, SYCP-3, etc., and there has been a spirited debate about how the two groups of proteins are organized relative to each other [6], [27]–[32], [37]–[42]. For Drosophila, the distinction would seem artificial at this point. The only proteins identified thus far that localize to the LEs – SMC1, SMC3, C(2)M, ORD, Nipped-B and SOLO – are all either cohesins or cohesion proteins with very close links to cohesins, and therefore seem likely to be components of the cores [30], [43], [45], [53], [60]–[63].

Our working model is that SOLO and ORD function as subunits of a cohesin complex that is distributed along the chromosome arms of all germ cells and likely provides cohesion between the sister chromatid axes. We do not dismiss the possibility that SOLO/ORD cohesin maintains cohesion in the chromatin loops as well but evidence has been presented that SMC cohesins are mostly confined to the axes in Drosophila germ cells [43]. In meiotic cells these arm cohesins condense along with C(2)M-cohesin (and perhaps other complexes) and assemble into continuous cores that underpin synapsis and SC formation. SOLO and ORD are unlikely to be components of different cohesin complexes since core stability was no worse when both ORD and SOLO were absent than when just SOLO was absent (Figures 5 and S7). Thus cores may consist of two cohesin complexes, one anchored by C(2)M and one anchored by ORD and SOLO. Additional cohesin complexes involving mitotic cohesins such as RAD21 might be present as well.

### SOLO and structure/assembly/maintenance of chromosome cores

Our observation that pro-oocytes with fragmented, patchy or no SMC1 and C(3)G staining are abundant even at the earliest stages of pachytene in *solo* mutants could indicate a requirement for SOLO in assembly of cores. In addition, the progressive degeneration of cores throughout early and mid-pachytene in *solo* mutants might indicate a possible role in core maintenance as suggested for *ord* [30], [43]. A role of SOLO in core assembly seems unlikely. Full-length cores can be assembled in the absence of SOLO or of both ORD and SOLO (Figures 5 and S7) [30], [43]. However, no cores are assembled in the absence of C(2)M, suggesting that C(2)M is the motor for assembly and that SOLO and ORD play passive roles [43], [45], [60]. A maintenance function is plausible but not especially compelling since it doesn't relate in any direct way to the primary function of SOLO. In our model, SOLO is a subunit of arm cohesin complexes that become assembled into cores in meiotic cells. This would make SOLO a structural component of WT cores and its absence would be expected to compromise core structure in one of two ways. First, cores assembled with abnormal (i.e., SOLO-deficient) cohesins might be less stable than WT cores and prone to breakage or disassembly. Second, exclusion of deficient cohesins from core assembly would likely lead to monolithic cores which might lack important structural or functional properties such as flexibility or ability to complete exchanges with homologous cores.

A major strength of this hypothesis is that it does not require a fundamentally different explanation for the *solo* and *ord* phenotypes, just a difference in degree of instability of the cores. If absence of SOLO is for some reason more destabilizing than absence of ORD, then it could trigger core degeneration at earlier stages of meiosis. One way this could work is based on our proposal that SOLO and ORD are subunits of the same cohesin complex. The effect of loss of a subunit on complex stability depends on the specific role of that subunit. For example, absence of the kleisin subunit is more destabilizing for conventional cohesin than absence of the SA subunit.

In *solo* mutants, all of the assembled cores in early pachytene must be defective but actual fragmentation and dissolution does not begin until later in pachytene in some cells. In other cells, dissolution is already complete in region 2a. This suggests that the defect creates a fragile state and that onset of degeneration may require a stressful event of some kind to trigger it, as suggested for *ord* [43]. The cell-to-cell variability in phenotype could reflect stochastic variation in degree of fragility, or perhaps cell-to-cell variation in the numbers or intensity of stressors.

### The role of SOLO in recombination

SOLO is required for completion of DSB repair on the normal schedule although the repair delay is brief compared with the delays caused by mutations in components of the DSB repair pathway (36,68–71). Mutations in other *Drosophila* chromosome core components such as *c(2)M* and *ord* have no effect at all on DSB repair (30,36,68). This is somewhat surprising in light of the often severe DSB repair defects seen in cohesin mutants in other eukaryotes (2,7–9,13,14). Additional studies will be required to determine if the transient repair delay in *solo* mutants contributes to its recombination phenotype.

Our data indicate that SOLO promotes homolog exchange and suppresses SCE (Tables 3–5). As SCE and homolog exchange are alternative pathways for DSB repair, suppressing SCE is likely to promote homolog exchange; direct molecular analysis of recombination intermediates in yeast confirms this [23], [24]. We conclude that a major role of SOLO in recombination is to regulate homolog bias, although this does not preclude SOLO acting in other ways to promote homolog exchange.

How might SOLO regulate homolog bias? There is a bit of a conundrum here: the primary function of SOLO is cohesion and although cohesion is very effective at promoting DSB repair, it does so by promoting SCE, presumably by reinforcing sister chromatid proximity [81]. REC8 becomes depleted around crossover sites presumably because it promotes SCE [82]. Moreover, in yeast, *rec8* mutations promote homolog bias, not sister bias [24]. Therefore, simply providing extra cohesion at a recombination site is more likely to inhibit homolog exchange than to promote it. An alternative is that the chromosome cores per se are responsible for suppressing SCE. Several recent models have postulated that AE/LEs serve as “barriers to sister chromatid repair” (BSCR) [28], [29]. This mechanism seems unlikely to apply to Drosophila because *c(2)M* mutations completely abrogate core assembly but do not de-repress SCE at all [45].

Our proposal is that SOLO/ORD-cohesin is an unconventional cohesin that is able to flexibly regulate cohesion in the context of meiotic recombination. It becomes enriched at future DSB sites, perhaps specifically at future crossover sites, during the synapsis initiation process, where it regulates the cohesive status of chromatids involved in the recombination reaction to promote inter-homolog exchanges. For example, relaxation of cohesion between the broken chromatid and its sister may be necessary to allow a homology search and inter-homolog strand invasion [24]. We speculate that ORD/SOLO-cohesin is able to rapidly switch to a “cohesion-off” mode in response to local signaling related to DSB or recombination intermediate status. In doing so, ORD/SOLO-cohesin might be able to promote homolog exchange locally while still maintaining cohesion globally.

In conclusion, SOLO is a meiotic cohesion protein with major roles in centromere cohesion, chromosome core integrity and homolog bias. It is enriched at centromeres and chromosome cores and interacts with the SMC1 cohesin subunit. Further investigation of SOLO's meiotic functions is expected to provide insight into the roles of cohesion in inter-homolog interactions.

## Materials and Methods

### Fly strains and culture methods

The *solo* mutants used in this paper were described previously [53]. *solo^Z2-0338^*, *solo^Z2-0198^* and *solo^Z2-3534^* are single-base substitutions predicted to insert stop codons in the SOLO coding sequence and truncate the proteins at amino acid positions 173, 387 and 1010 (out of 1031), respectively [53]. All three alleles are considered to be functionally null with respect to chromosome segregation. Although a closely-linked semi-lethal mutation has thus far prevented accurate measurement of NDJ in *solo^Z2-3534^* homozygotes, both male and female sex chromosome NDJ frequencies in the other two homozygotes and in all three hemizygotes are statistically indistinguishable and consistent with random chromatid segregation [53] (Table1, unpublished data). The *b vas^7^* stock was obtained from M. Ashburner (Cambridge University, England). The X chromosome mapping stock *y pn cv m f.y^+^/FM7c* was provided by K. McKim (The state University of New Jersey). *ord^5^* and *Df(2R)WI370* were donated by S.E. Bickel (Dartmouth College). The *GFP::VAS* transgenic line was provided by P. Lasko (McGill University). Other flies were from the Bloomington Drosophila Stock Center at Indiana University. Unless otherwise specified, the females being tested were crossed singly to two males in shell vials. All flies were maintained at 23°C on standard cornmeal molasses medium. Parents were removed from the vial on day 10 and progeny were counted between day 13 and day 22.

### Assaying NDJ and recombination on the X and 2nd chromosomes

The methods for analyzing NDJ and recombination on the X and second chromosomes are explained and illustrated in Figures S1 and S2 and in Tables 1–4 and S1.

### Viability correction for chromosome 2 NDJ cross (Table 2)

To accurately estimate the relative frequencies of sister and homolog NDJ, it is necessary to correct for the reduced viability of the sister NDJ classes which are homozygous for most or all of chromosome 2, relative to the homolog NDJ classes, which are heterozygous. The viability test was based on recoveries of the homozygote and heterozygote progeny classes from two crosses: *solo^Z2-0198^ cn bw/b vas^7^* males crossed to *solo^Z2-0198^ cn bw/Cy* females and *solo^Z2-0198^ cn bw/b vas^7^* males crossed to *b vas^7^/Cy* females. The viabilities of *b vas^7^* and *solo^Z2-0198^ cn bw* homozygotes were found to be 51.76% and 63.49%, respectively, compared to their heterozygous siblings (*b/cn bw*). Plugging the decimal versions of those correction factors into the formula for %S NDJ gives %S = 100×(144/0.5176+106/0.6349+37)/((144/0.5176+106/0.6349+37)+(1012+36)) = 32%.

### Assaying sister chromatid exchange

*R(1)2, y^1^ f^1^*/*B^S^Yy^+^* males were crossed to *Df(2L)A267, b cn bw*/*CyO, cn* females. The *R(1)2, y^1^ f^1^*/+; *Df(2L)A267, b cn bw*/+ F1 female progeny were crossed to *y w*/Y; *solo, cn bw*/CyO, *b cn* males to generate F2 *R(1)2, y^1^*/y w; *Df(2L)A267, b cn bw*/*solo, cn bw* females and sibling control *R(1)2, y^1^*/*y w*; +/CyO, *b cn* females. These F2 females were crossed to *w^1118^*/Y males and their progeny scored for the ring-X (w+) and rod-X (w). The crosses were carried out without an X chromosome balancer to enable estimation of SCE frequencies under conditions in which both homolog and sister chromatid exchanges were free to occur. The ring-X chromosome was tracked using the *y/y^+^* marker in the F1 cross and the *w/w^+^* marker in the F2 (test) cross. The *y^1^* allele on the ring-X chromosome is recombinationally inseparable from the centromere, and *w*, which is 1.5 cM from *y*, does not recombine with *y* at appreciable rates in ring/rod heterozygotes where only double exchanges can be recovered (unpublished data). In the F1 cross, *cn* was used as a proxy for *Df(2L)A267. solo/Df* F2 females were sorted by Cy cn^+^ phenotype and verified (or not) on the basis of fertility and NDJ. Only regular (disjunctional) progeny were used to calculate the ring/rod recovery ratio.

### Construction of FH::SOLO fusion clones and generation of transgenic flies

pENTR-Ntag-SOLO entry vector [53] was recombined into Gateway P-element vector pPFH (Drosophila Genomics Resource Center (BDGC)), generating the germ line transformation vector *P{w^+mC^ UASp-FH::SOLO}*, which contains tandem 3XFLAG and 3XHA tags at the N-terminus of SOLO fusion protein. The construct was transformed into *w^1118^* flies (BestGene Inc.). Transgenes were mapped by standard methods and tested for ability to suppress X chromosome NDJ in *solo* females when expressed with the *nos-GAL4::VP16* driver [83] (see Table S1, lines 4 and 5).

### Immunoprecipitation of FH::SOLO

FH::SOLO expression was induced by *nos-GAL4::VP16* in Drosophila females and 100 pairs of ovaries were collected with 1X PBS (pH 7.4). Ovaries of *y w* and transgenic flies were lysed using 500 µL of NP40 Cell Lysis Buffer (Invitrogen). The lysates were centrifuged at least 4 times each at 13,000 g for 10 minutes to remove tissue debris and the supernatants were used for Western blots and immunoprecipitations. Before immunoprecipitating, lysates were first pre-cleared with rabbit serum. 4 µL of rabbit serum (159 mg/ml, Sigma) were added to the 500 µL lysates and rocked for 1 hr at 4°C, then the lysates with rabbit serum were added to 100 µl of protein A agarose beads (Invitrogen) which had been washed 5 times with wash buffer (1 mM PMSF, 1 mM DTT, 1X PI (Protease Inhibitor (Roche)), 10% glycerol, 10 mM NaCl, 1X PBS, pH 7.4) rocking for 30 minutes at 4°C.

To immunoprecipitate FH::SOLO with anti-SMC1, 50 µl of pre-cleared lysates were incubated with 20 µl of anti-SMC1 rabbit antibody (1.03 mg/ml) or rabbit serum (1.06 mg/ml, diluted from original serum) and IP solution (1 mM PMSF, 1 mM DTT, 1X PI (Protease Inhibitor (Roche)), 10% glycerol, 1X PBS, pH 7.4) rocking for 4 hrs. The lysates with anti-SMC1 antibody or serum were then added to 80 µL of washed protein A agarose beads and rocked overnight in a cold room at 4°C.

To immunoprecipitate SMC1 by FH::SOLO, 50 µl of lysate pre-cleared with mouse serum and protein G agarose beads (similar procedure to rabbit serum) were incubated with 30 µL of anti-FLAG M2 (1 mg/ml, Sigma) or mouse serum (1.10 mg/ml, diluted from original serum) and the IP solution was rocked for 4 hrs. Lysates with anti-SMC1 antibody or serum were then added to 100 µl of washed protein G agarose beads and rocked overnight in a cold room (4°C).

After IP, lysates/antibody or serum/IP solutions/beads were centrifuged and beads were washed 6× times with wash buffer. 30 µL of loading buffer were added to the beads and heated to release protein binding to the beads.

### Western blot

The lysates from FH::SOLO and *y w* flies that were used in Western blot to test antibody specificity and the released solutions (from IP experiment) were run in 8% SDS-PAGE Acr/Bis electrophoresis. FH::SOLO was detected by using anti-FLAG M2 antibody (1∶1000, Sigma) and goat anti-mouse HRP-conjugated (1∶1000, Chemicon) with Supersignal West Pico (Pierce). SMC1 was detected by using anti-SMC1 (1∶200, rabbit) and goat anti-rabbit HRP-conjugated (1∶2000, JacksonImmuno) with Supersignal West Pico (Pierce).

### Immunostaining

Newly eclosed females were fattened 1–3 days in vials with yeast paste and males and then ovaries were dissected in 1X PBS (pH 7.4). Immunostaining of whole-mount ovarioles was performed according to Page and Hawley [34]. After immunostaining, ovaries were separated into individual ovarioles and transferred to slides and mounted with Prolong Antifade reagent (Invitrogen). *UASp-Venus::SOLO* expression was induced by *nos-GAL4::VP16* and fluorescent signals were detected in the FITC channel or detected by anti-GFP antibody. Egg chambers were staged according to Matthies et al. [84]. Chromosome spreads were performed according to Webber et al. [30].

### Identification and staging of pro-oocytes and oocytes in cytological analysis

For WT germaria, pro-oocytes and oocytes in pachytene were identified by full-length C(3)G nuclear staining and enriched cytoplasmic ORB staining. For *solo* germaria, pro-oocytes and oocytes were identified by enriched cytoplasmic ORB staining, except in Figure S6 where C(3)G staining was used. In that figure, the “no-staining” category was not scored. For *solo* pro-oocytes in region 2a with abnormal C(3)G staining, the ORB-enrichment criterion ensured that zygotene cysts were not inadvertently included in the scoring. Even without ORB staining, however, zygotene nuclei could usually be distinguished from the defective pachytene nuclei by C(3)G staining. The C(3)G foci are usually smaller and more uniform in size in zygotene than the “spotty” staining in pachytene, and lengthy linear fragments are never seen in zygotene. Staging (regions 2a, 2b, and 3) was based on position of cysts in the germarium (see Figure 1) and/or shape of cysts (rounded in region 2a, flattened in region 2b). Oocytes in egg chambers were identified by ORB enrichment, C(3)G staining, nuclear size (smaller than polyploid nurse cell nuclei) and/or position in cyst (posterior). For scoring of γ-H2Av foci, pro-oocytes and oocytes were identified by enriched ORB staining. Pro-nurse cell nuclei were not scored.

### Scoring centromere numbers in germ cells

Linear C(3)G staining was used to identify pachytene pro-oocytes and oocytes from the 2a region of WT and *solo* germaria. Nuclear boundaries were established based on margins of DAPI and C(3)G staining. Nuclei with overlapping DAPI or C(3)G staining were not used for scoring. Only non-overlapping CID spots were scored as separate spots. Size and brightness of CID spots was not considered.

### Antibodies used

Primary antibodies used : 1∶500 anti-C(3)G (mouse monoclonal and guinea pig polyclonal antibody (provided by R.S. Hawley), 1∶500 rabbit anti-GFP polyclonal antibody (Invitrogen), 1∶800 rabbit anti-CID polyclonal antibody (Active Motif), 1∶200 anti-SMC1 rabbit polyclonal antibody [53], [54], 1∶5000 rabbit anti-γ-H2Av antibody (Rockland), 1∶3000 anti-VASA antibody (P. Lasko), 1∶150 anti-ORB (6H4 and 4H8, monoclonal, Developmental Studies Hybridoma Bank (DSHB)). Secondary antibodies (IgGs) used: Alexa Fluor 488 donkey anti-rabbit, Alexa Fluor 488 goat anti-guinea pig, Alexa Fluor 555 donkey anti-mouse, Alexa Fluor 555 donkey anti-rabbit, Alexa Fluor 647 donkey anti-mouse (Invitrogen).

### Microscopy and image processing

All images were collected using an Axioplan (ZEISS) microscope equipped with an HBO 100-W mercury lamp and high-resolution CCD camera (Roper). Image data were collected and merged using MetaMorph Software (Universal Imaging Corporation). Adobe Photoshop CS2 and Illustrator CS2 were used to process images. Each image in the immunofluorescence figures came from a sum projection of 3D deconvolved z-series stacks. All images from WT and mutants were exposed for equal periods and deconvolved and processed identically.

## Supporting Information

Figure S1 Measurement of NDJ and recombination on X chromosomes. *Dp(1;1)sc^v1^, y pn cv m f.y^+^/y; solo cn bw/Df(2L)A267, b cn bw* females were crossed with *YSX.YL, In(1)EN, y B/Y* males *(X∧Y/Y)*. The X genotype of the females is shown in (A). The *yellow* ^+^ (*y^+^*) marker on *Dp(1;1)sc^v1^* is carried on a duplication on XR and is inseparable from the centromere. The regular progeny from this cross are B^+^ males and B females. The B^+^ males were used to score recombination (Table 3). NDJ yields B^+^ females that result from diplo-X eggs fertilized by *Y* sperm and y B males that result from nullo-X eggs fertilized by *YSX.YL, In(1)EN, y B* sperm. Diplo-X eggs carry either two homologous centromeres (B) or two sister centromeres (C). They can be distinguished by their genotypes at the *f (forked)* and *y^+^* loci that flank the centromere region. y^+^ f^+^ progeny result from homolog NDJ while y^+^ f and y f^+^ progeny result from sister chromatid NDJ. Additional classes of homolog and sister NDJ could result from recombination between *f* and the centromere (such as *y f* sister NDJs) but are not pictured because no *f-y* ^+^ recombinants were recovered among the progeny of diplo-X eggs in any the *solo* mutant crosses.
(PDF)

Figure S2 Analysis of NDJ and recombination on chromosome 2. (A) *solo, cn bw/b vas^7^* females were used for both the NDJ and recombination crosses. *vas^7^* is a recessive *vasa* allele [87] that is also a null allele of *solo* (data not shown). See Table 4 legend for a description of the recombination cross and analysis. To test for chromosome 2 NDJ and to estimate the relative frequencies of sister and homolog NDJ, *solo, cn bw/b vas^7^* females were crossed singly to two *C(2)EN, bw sp* males. *C(2)EN* males generate only diplo-2, *bw sp* and nullo-2 sperm, so viable, euploid progeny are produced only from fertilization by reciprocally aneuploid eggs that result from chromosome 2 NDJ in the female. (B and C) Diplo-2 eggs can carry centromeres either from two homologous or two sister chromatids, so can be used to determine the relative frequencies of homolog and sister NDJ. As shown, these classes can be discriminated by their genotypes with respect to the *b* and *cn* markers that flank and are near the centromere. The left panels show the patterns in the absence of crossing over; the right panels show the patterns if there is recombination between the *cn* and *bw* loci. Note: the top two genotypes in panel C cannot be distinguished phenotypically but the bottom two genotypes can be. Recombination between *b* and *cn* would yield additional genotypes but are not shown because no such events were recovered among the progeny of diplo-2 eggs in either cross.
(PDF)

Figure S3 UPS-SOLO::Venus localizes to chromosome arms in pro-oocytes and pro-nurse cells. Chromosome spread preparations from (A) a pro-oocyte and (B) a pro-nurse cell from *Df(2L)A267/solo^Z2-0198^; {UPS-SOLO::Venus}* females. SOLO::Venus was detected by native fluorescence. SC was visualized by C(3)G staining and DNA was stained with DAPI. Scale bars: 5 µm. SOLO formed bright foci (probably centromeres) and patchy arm staining in both pro-oocytes (shown by C(3)G) and pro-nurse cells (showing no C(3)G staining).
(PDF)

Figure S4 Defective synaptonemal complexes in late pachytene in *solo* mutants. Each image comes from a sum projection of 3D deconvolved z-series of stage 4 egg chambers. SC was visualized by anti-C(3)G staining (green) and DNA was stained with DAPI (red). Scale bar: 5 µm. C(3)G staining was extensive in an oocyte nucleus in a WT egg chamber. However, in *solo* (*solo^Z2-0198^*/*Df* and *solo^Z2-3534^*/*Df*) mutants, C(3)G staining was much reduced and present only as separate foci.
(PDF)

Figure S5 Effects of GFP::VAS expression on ORB expression and distribution in *solo* germaria. VASA is a cytoplasmic protein expressed in all germ cells. GFP::VAS is a full-length VASA cDNA tagged with GFP and expressed under control of the native *vasa* promoter [65]. GFP::VAS has been shown to rescue fertility of sterile *vas* mutants [65]. Scale bar: 5 µm. (A, B) VASA and ORB expression in WT and *solo* germaria. WT (A) and *Df(2L)A267/solo^Z2-0198^* (B) germaria were stained with anti-VASA and anti-ORB antibodies. DNA was visualized by DAPI. Anti-VASA staining patterns and intensity did not visibly differ between WT and *solo* germaria. However, the *solo* germarium was distinctly thinner than the WT germarium and contained fewer anti-VASA stained cells and far fewer anti-ORB stained cells than the WT germarium. Nevertheless, ORB distribution in ORB-positive cysts appeared normal. (C) Effects of GFP::VAS on ORB expression and localization in *solo* germaria. GFP::VAS was detected by native fluorescence in *Df(2L)A267/solo^Z2-0198^; GFP::VAS/+* germarium. ORB was detected with anti-ORB antibody. DNA was visualized by DAPI. Germarium was much fatter with many more anti-VASA and anti-ORB stained cells than the *solo* germarium in (B). ORB distribution within cysts appeared normal.
(PDF)

Figure S6 Expression of *GFP::VAS* in *solo* germarium does not affect C(3)G staining pattern. SCs were visualized by anti-C(3)G antibody. DNA was stained with DAPI. Scale bars: 5 µm. (A) WT. (B) *Df(2L)A267/solo^Z2-0198^*. (C) *Df(2L)A267/solo^Z2-0198^; GFP::VAS/+*. Germarium in (C) shows many more pro-oocytes than germarium in (B) due to increased VASA but C(3)G staining patterns remained abnormal (arrowheads). (D) Quantification of C(3)G phenotypes of region 2a and 2b pro-oocytes from *Df(2L)A267/solo^Z2-0198^* and *Df(2L)A267/solo^Z2-0198^; GFP::VAS/+* respectively. Only C(3)G-stained pro-oocytes were scored.
(PDF)

Figure S7 C(3)G phenotypes in *ord* and *solo ord* double mutants. SCs were visualized by anti-C(3)G antibody. Pro-oocytes and oocytes were identified by enriched ORB staining (not shown). DNA was stained with DAPI. Scale bars: 5 µm. (A) C(3)G staining in *ord^5^/Df(2R)WI370* germarium showed normal staining in region 2a, fragmented staining in region 2b and minimal staining in region 3. (B) C(3)G staining in *solo^Z2-0198^ ord^5^/solo^Z2-3534^ ord^Z2-5736^* double mutant showed defective staining throughout regions 2a-3, similar to the pattern in *solo* single mutants (compare to Figures 5B and 5C). See Figure 5D for quantification.
(PDF)

Figure S8 Quantification of γ-H2Av foci in *solo* germaria. The graphs show the percentages of pro-oocytes and oocytes exhibiting different numbers of γ-H2Av foci and short stretches per nucleus. Pro-oocytes and oocytes from *solo* mutant females (*solo^Z2-3534^*/*Df*) and WT sibling controls were identified by enriched ORB staining and by their relative positions within germarium. (A) Region 2a: 58 WT and 44 *solo* nuclei were scored. (B) Region 2b: 38 WT and 56 *solo* nuclei were scored. (C) Region 3: 33 WT and 24 *solo* nuclei were scored.
(PDF)

Figure S9 Effect of GFP::VAS expression on γ-H2Av staining in *solo* pro-oocyte and oocyte nuclei. Female genotype was *Df(2L)A267/solo^Z2-0198^; GFP::VAS/TM6*. DSBs were stained by anti- γ-H2Av antibody. Pro-oocytes and oocytes were identified by enriched staining with anti-ORB antibody. DNA was visualized with DAPI. Scale bars: 5 µm. (A) γ-H2Av in germarium. Note the foci in the region 3 oocyte nucleus. (B) γ-H2Av in a stage 2 egg chamber that is from the same ovariole as in (A). Foci are absent in the oocyte although some foci are still present in nearby nurse cells nuclei.
(PDF)

Table S1 Results of SOLO transgene NDJ rescue experiments. ^a^The indicated females were crossed to *Y^S^X.Y^L^, In(1)EN, y B* males to measure X chromosome NDJ. *Df* represents *Df(2L)A267*. *nos-GAL4* represents *nos-GAL4::VP16*. *UPS-SOLO* represents *{UPS-SOLO::Venus}*. The numbers in parentheses in lines 6–8 represent the copy number of *UPS-SOLO*. The generation of *{UASp-Venus::SOLO}*, *{UASp-SOLO::Venus}* and *{UPS-SOLO::Venus}* were described in [53]. ^b^% NDJ = 100×2× (B^+^ females+y B males)/(N+B^+^ females+y B males). ^c^N = total number of progeny. Note: the incomplete rescue of X-X NDJ by the *UPS-SOLO::Venus* construct (lines 6–8) was not due to the C-terminal location of the Venus tag. The same SOLO::Venus protein fully rescued NDJ when expressed under control of the *nos-GAL4::VP16* driver (line 3). In tests for rescue of X-Y NDJ in *solo* males, NDJ was reduced to 5.8%, 3.25% or 1.8% with one, two or three copies, respectively, of *{UPS-SOLO::Venus}* (unpublished data).
(PDF)

Table S2 Partial rescue of *solo* female fertility by GFP::VAS. The indicated females were crossed singly to two *y w* males to measure fertility and X chromosome NDJ. NDJ was estimated only from the patriclinous sons (derived from nullo-X eggs and X sperm and denoted by “n”) because the matriclinous daughters were indistinguishable from the regular daughters. % NDJ = 100×4n/(N+2n).
(PDF)
