# Supplementary figures and images for: The Cohesion Protein SOLO Associates with SMC1 and Is Required for Synapsis, Recombination, Homolog Bias and Cohesion and Pairing of Centromeres in Drosophila Meiosis
Source: PLoS Genet. 2013 Jul 18;9(7):e1003637. doi: 10.1371/journal.pgen.1003637 (PMC3715423; doi:10.1371/journal.pgen.1003637)

A.Homologous X chromosomes

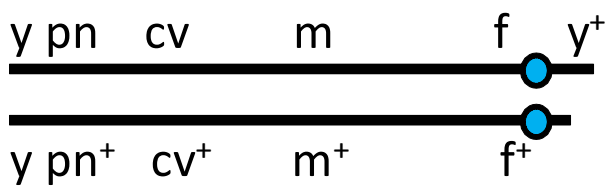

B.Homolog NDJ

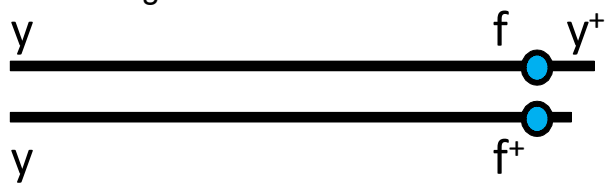

C. Sister chromatid NDJ

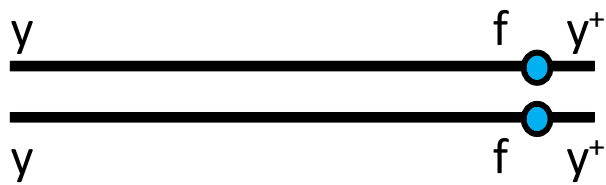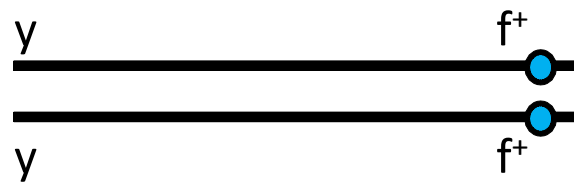

Figure S1

Supplement: Figure S1 — Measurement of NDJ and recombination on X chromosomes. Dp(1;1)scv1, y pn cv m f.y+/y; solo cn bw/Df(2L)A267, b cn bw females were crossed with YSX.YL, In(1)EN, y B/Y males (X∧Y/Y). The X genotype of the females is shown in (A). The yellow + (y+) marker on Dp(1;1)scv1 is carried on a duplication on XR and is inseparable from the centromere. The regular progeny from this cross are B+ males and B females. The B+ males were used to score recombination (Table 3). NDJ yields B+ females that result from diplo-X eggs fertilized by Y sperm and y B males that result from nullo-X eggs fertilized by YSX.YL, In(1)EN, y B sperm. Diplo-X eggs carry either two homologous centromeres (B) or two sister centromeres (C). They can be distinguished by their genotypes at the f (forked) and y+ loci that flank the centromere region. y+ f+ progeny result from homolog NDJ while y+ f and y f+ progeny result from sister chromatid NDJ. Additional classes of homolog and sister NDJ could result from recombination between f and the centromere (such as y f sister NDJs) but are not pictured because no f-y + recombinants were recovered among the progeny of diplo-X eggs in any the solo mutant crosses. (PDF) [file pgen.1003637.s001.pdf]

A. pro-oocyte

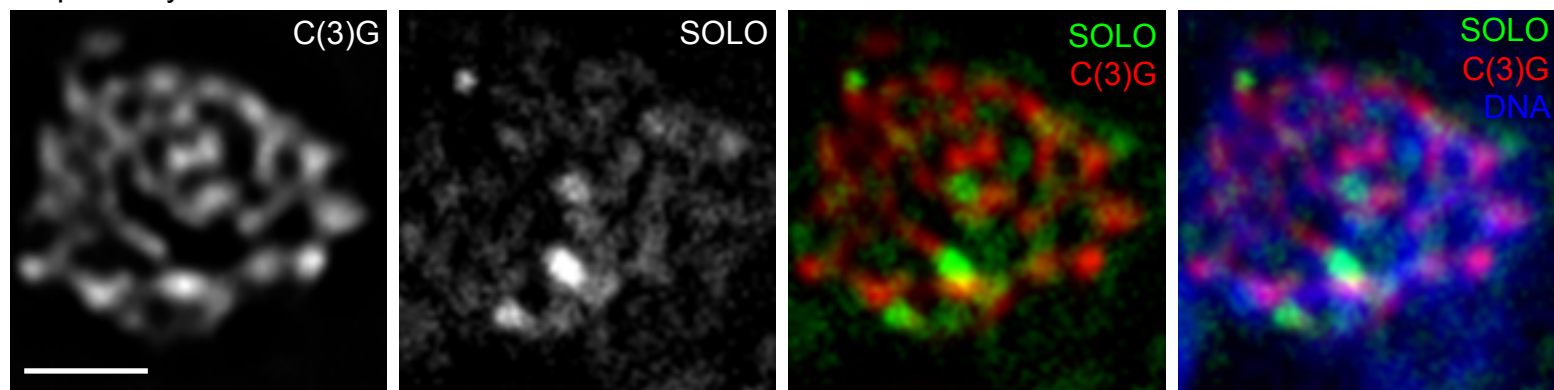

B. pro-nurse cell

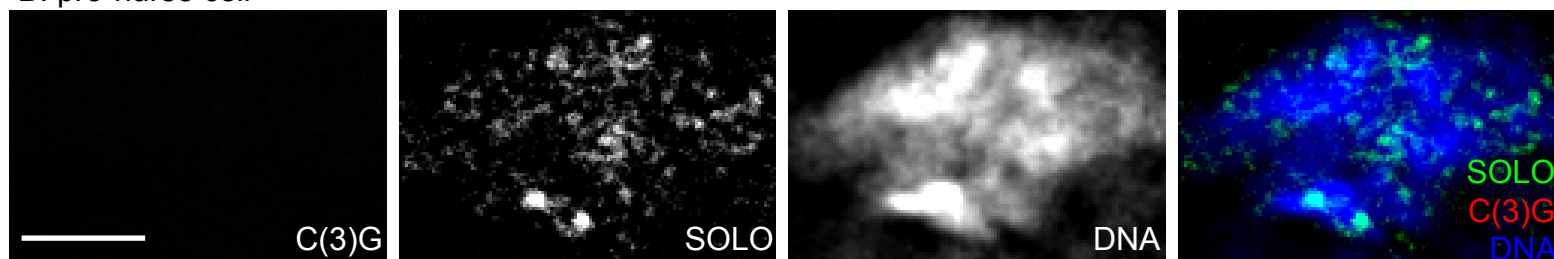

Figure S3

Supplement: Figure S3 — UPS-SOLO::Venus localizes to chromosome arms in pro-oocytes and pro-nurse cells. Chromosome spread preparations from (A) a pro-oocyte and (B) a pro-nurse cell from Df(2L)A267/soloZ2-0198; {UPS-SOLO::Venus} females. SOLO::Venus was detected by native fluorescence. SC was visualized by C(3)G staining and DNA was stained with DAPI. Scale bars: 5 µm. SOLO formed bright foci (probably centromeres) and patchy arm staining in both pro-oocytes (shown by C(3)G) and pro-nurse cells (showing no C(3)G staining). (PDF) [file pgen.1003637.s003.pdf]

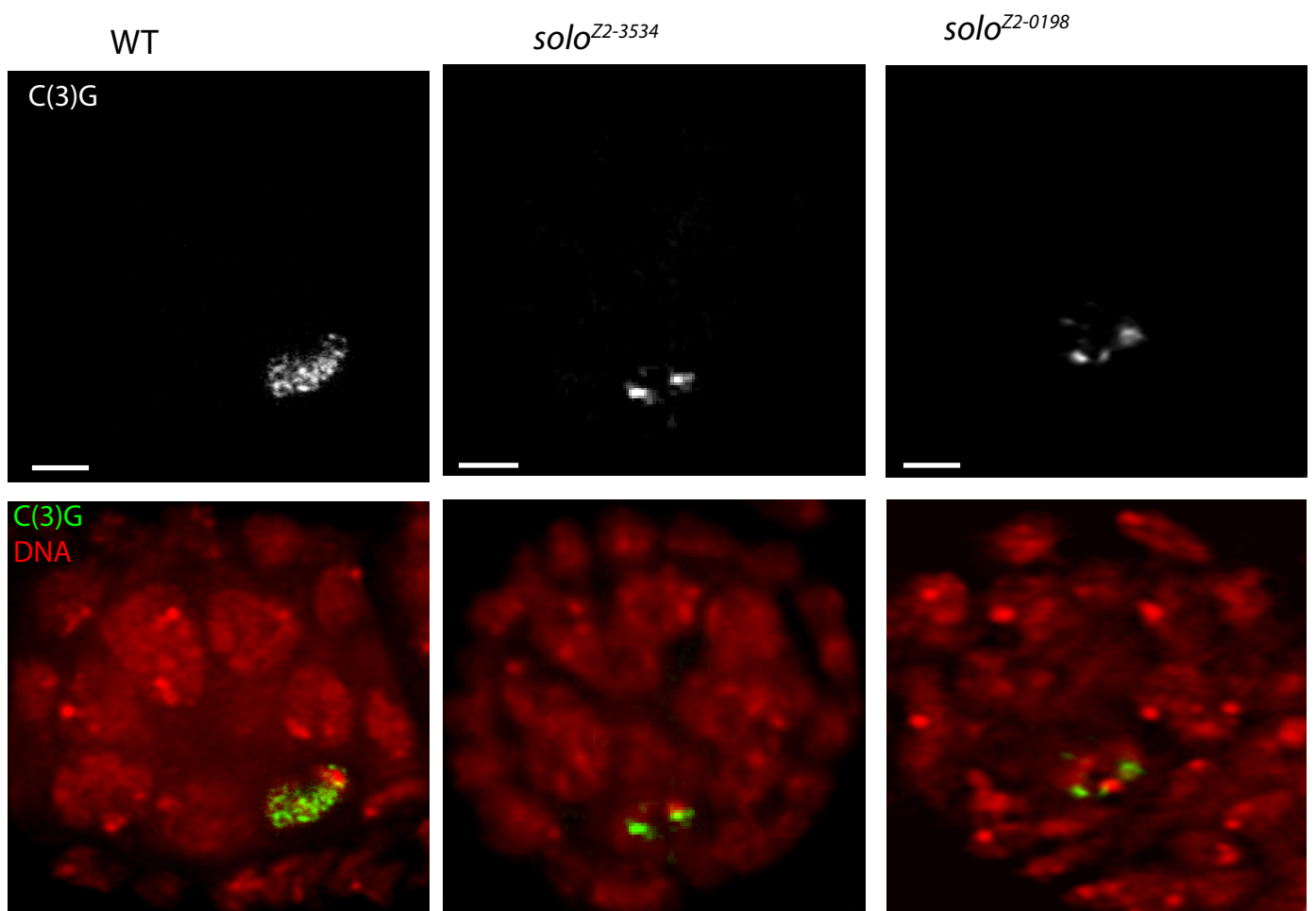

Figure S4

Supplement: Figure S4 — Defective synaptonemal complexes in late pachytene in solo mutants. Each image comes from a sum projection of 3D deconvolved z-series of stage 4 egg chambers. SC was visualized by anti-C(3)G staining (green) and DNA was stained with DAPI (red). Scale bar: 5 µm. C(3)G staining was extensive in an oocyte nucleus in a WT egg chamber. However, in solo (soloZ2-0198/Df and soloZ2-3534/Df) mutants, C(3)G staining was much reduced and present only as separate foci. (PDF) [file pgen.1003637.s004.pdf]

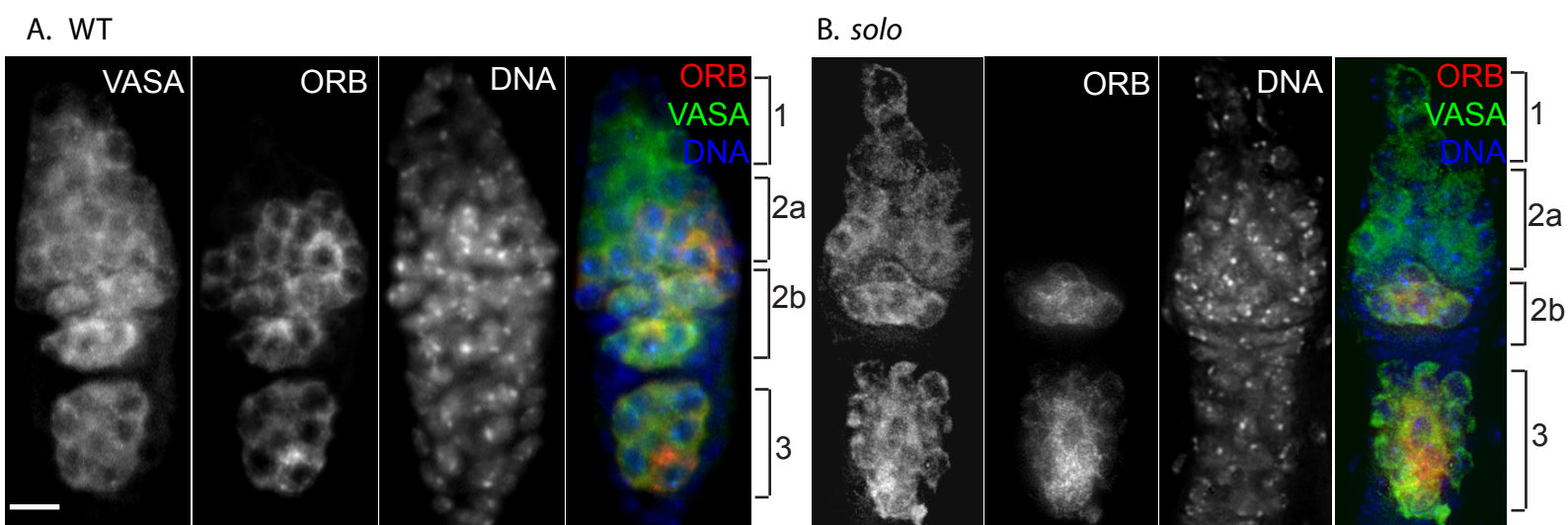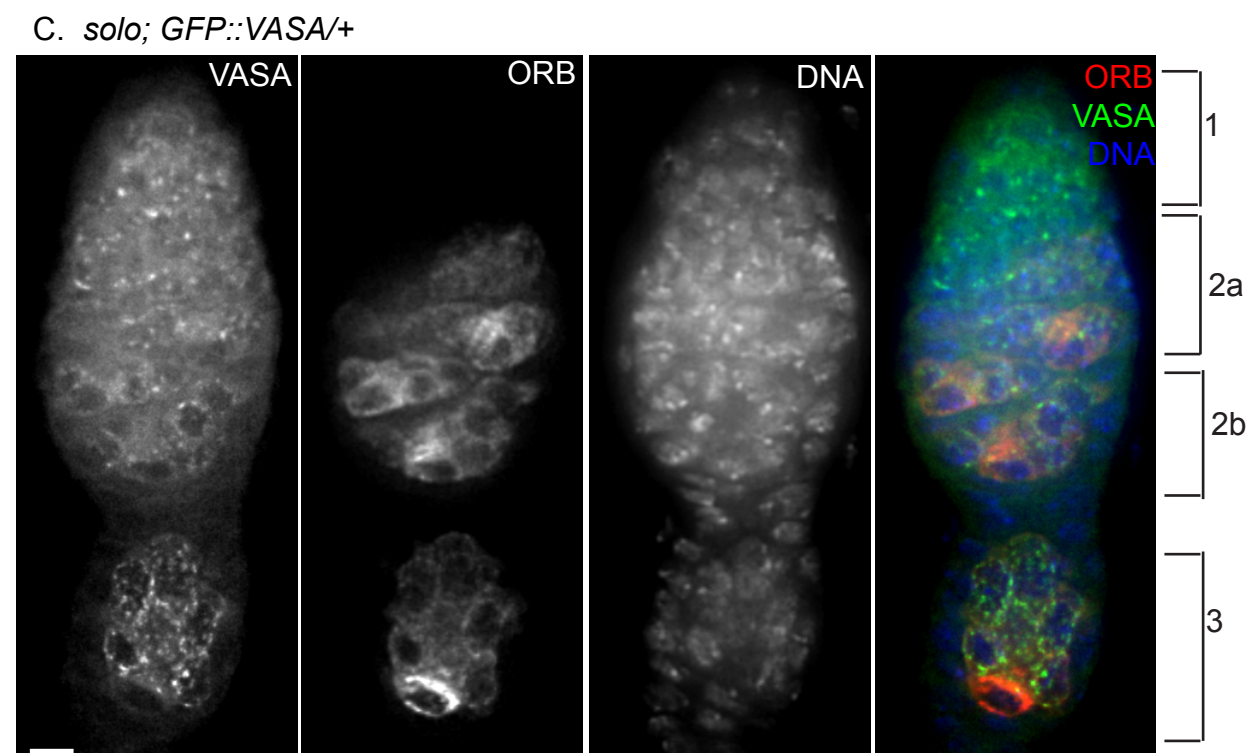

Figure S5

Supplement: Figure S5 — Effects of GFP::VAS expression on ORB expression and distribution in solo germaria. VASA is a cytoplasmic protein expressed in all germ cells. GFP::VAS is a full-length VASA cDNA tagged with GFP and expressed under control of the native vasa promoter [65]. GFP::VAS has been shown to rescue fertility of sterile vas mutants [65]. Scale bar: 5 µm. (A, B) VASA and ORB expression in WT and solo germaria. WT (A) and Df(2L)A267/soloZ2-0198 (B) germaria were stained with anti-VASA and anti-ORB antibodies. DNA was visualized by DAPI. Anti-VASA staining patterns and intensity did not visibly differ between WT and solo germaria. However, the solo germarium was distinctly thinner than the WT germarium and contained fewer anti-VASA stained cells and far fewer anti-ORB stained cells than the WT germarium. Nevertheless, ORB distribution in ORB-positive cysts appeared normal. (C) Effects of GFP::VAS on ORB expression and localization in solo germaria. GFP::VAS was detected by native fluorescence in Df(2L)A267/soloZ2-0198; GFP::VAS/+ germarium. ORB was detected with anti-ORB antibody. DNA was visualized by DAPI. Germarium was much fatter with many more anti-VASA and anti-ORB stained cells than the solo germarium in (B). ORB distribution within cysts appeared normal. (PDF) [file pgen.1003637.s005.pdf]

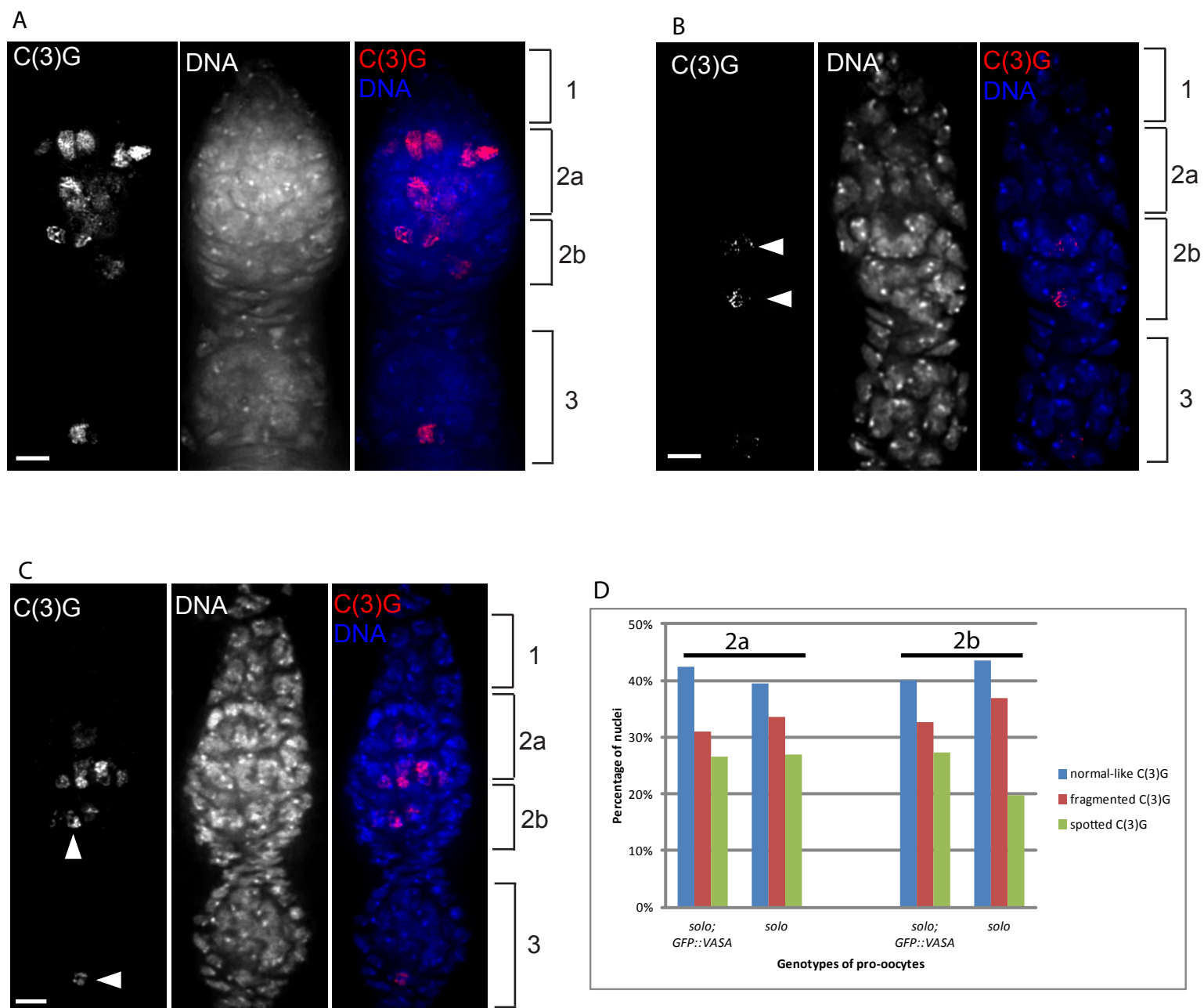

Figure S6

Supplement: Figure S6 — Expression of GFP::VAS in solo germarium does not affect C(3)G staining pattern. SCs were visualized by anti-C(3)G antibody. DNA was stained with DAPI. Scale bars: 5 µm. (A) WT. (B) Df(2L)A267/soloZ2-0198. (C) Df(2L)A267/soloZ2-0198; GFP::VAS/+. Germarium in (C) shows many more pro-oocytes than germarium in (B) due to increased VASA but C(3)G staining patterns remained abnormal (arrowheads). (D) Quantification of C(3)G phenotypes of region 2a and 2b pro-oocytes from Df(2L)A267/soloZ2-0198 and Df(2L)A267/soloZ2-0198; GFP::VAS/+ respectively. Only C(3)G-stained pro-oocytes were scored. (PDF) [file pgen.1003637.s006.pdf]

A. *ord*

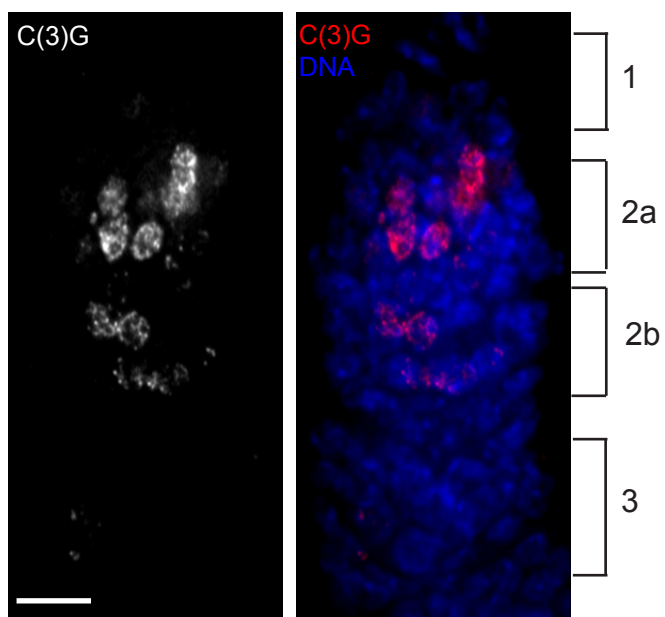

B. *solo ord*

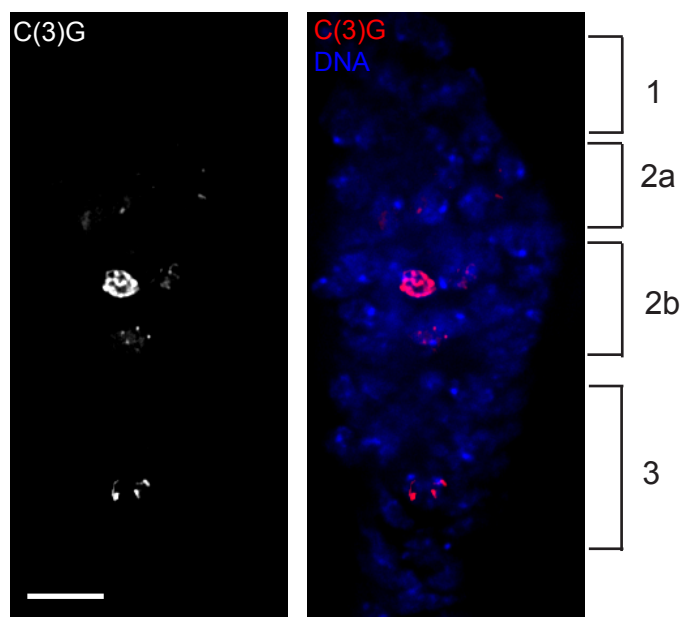

Figure S7

Supplement: Figure S7 — C(3)G phenotypes in ord and solo ord double mutants. SCs were visualized by anti-C(3)G antibody. Pro-oocytes and oocytes were identified by enriched ORB staining (not shown). DNA was stained with DAPI. Scale bars: 5 µm. (A) C(3)G staining in ord5/Df(2R)WI370 germarium showed normal staining in region 2a, fragmented staining in region 2b and minimal staining in region 3. (B) C(3)G staining in soloZ2-0198 ord5/soloZ2-3534 ordZ2-5736 double mutant showed defective staining throughout regions 2a-3, similar to the pattern in solo single mutants (compare to Figures 5B and 5C). See Figure 5D for quantification. (PDF) [file pgen.1003637.s007.pdf]

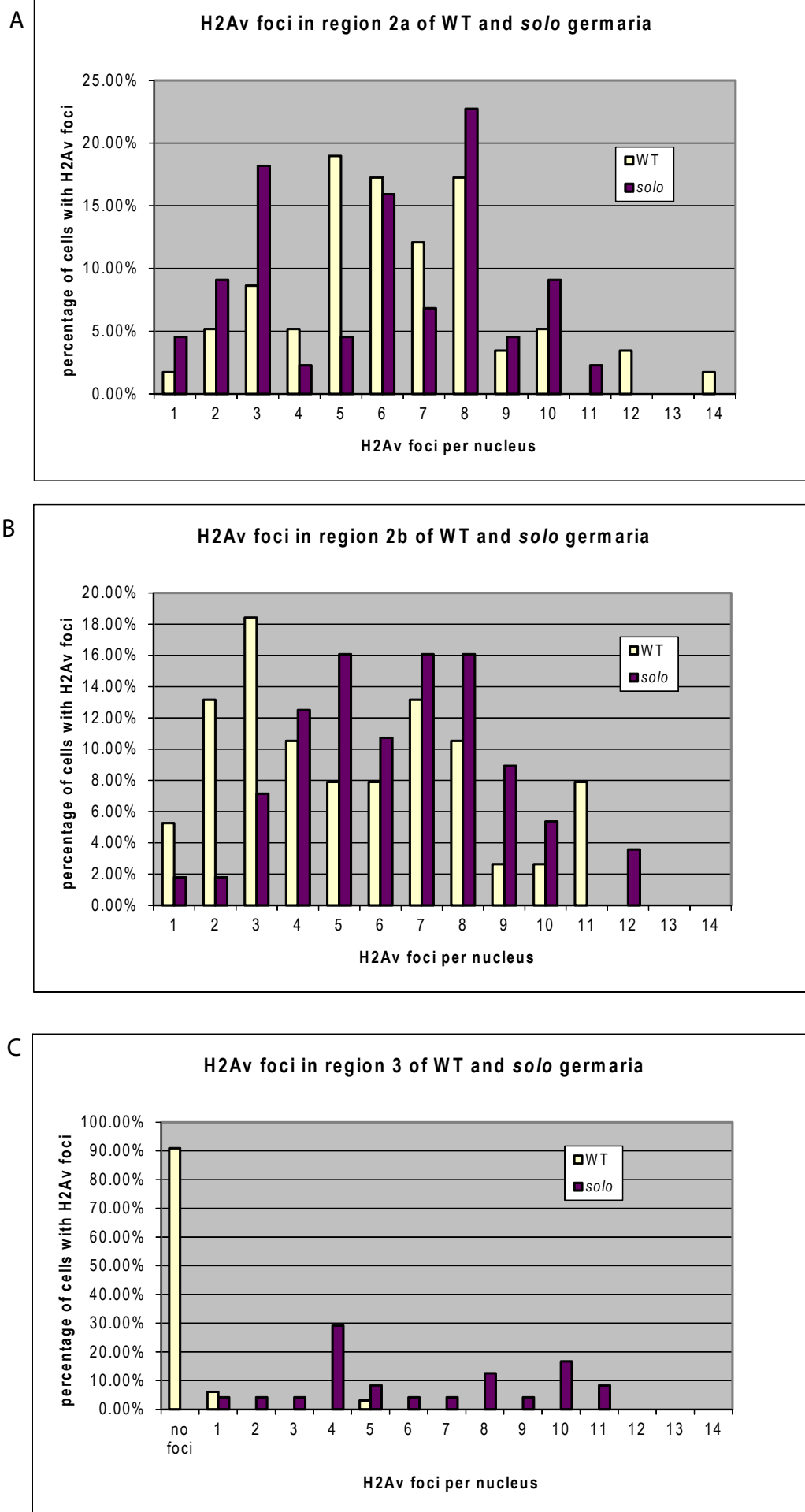

Figure S8

Supplement: Figure S8 — Quantification of γ-H2Av foci in solo germaria. The graphs show the percentages of pro-oocytes and oocytes exhibiting different numbers of γ-H2Av foci and short stretches per nucleus. Pro-oocytes and oocytes from solo mutant females (soloZ2-3534/Df) and WT sibling controls were identified by enriched ORB staining and by their relative positions within germarium. (A) Region 2a: 58 WT and 44 solo nuclei were scored. (B) Region 2b: 38 WT and 56 solo nuclei were scored. (C) Region 3: 33 WT and 24 solo nuclei were scored. (PDF) [file pgen.1003637.s008.pdf]

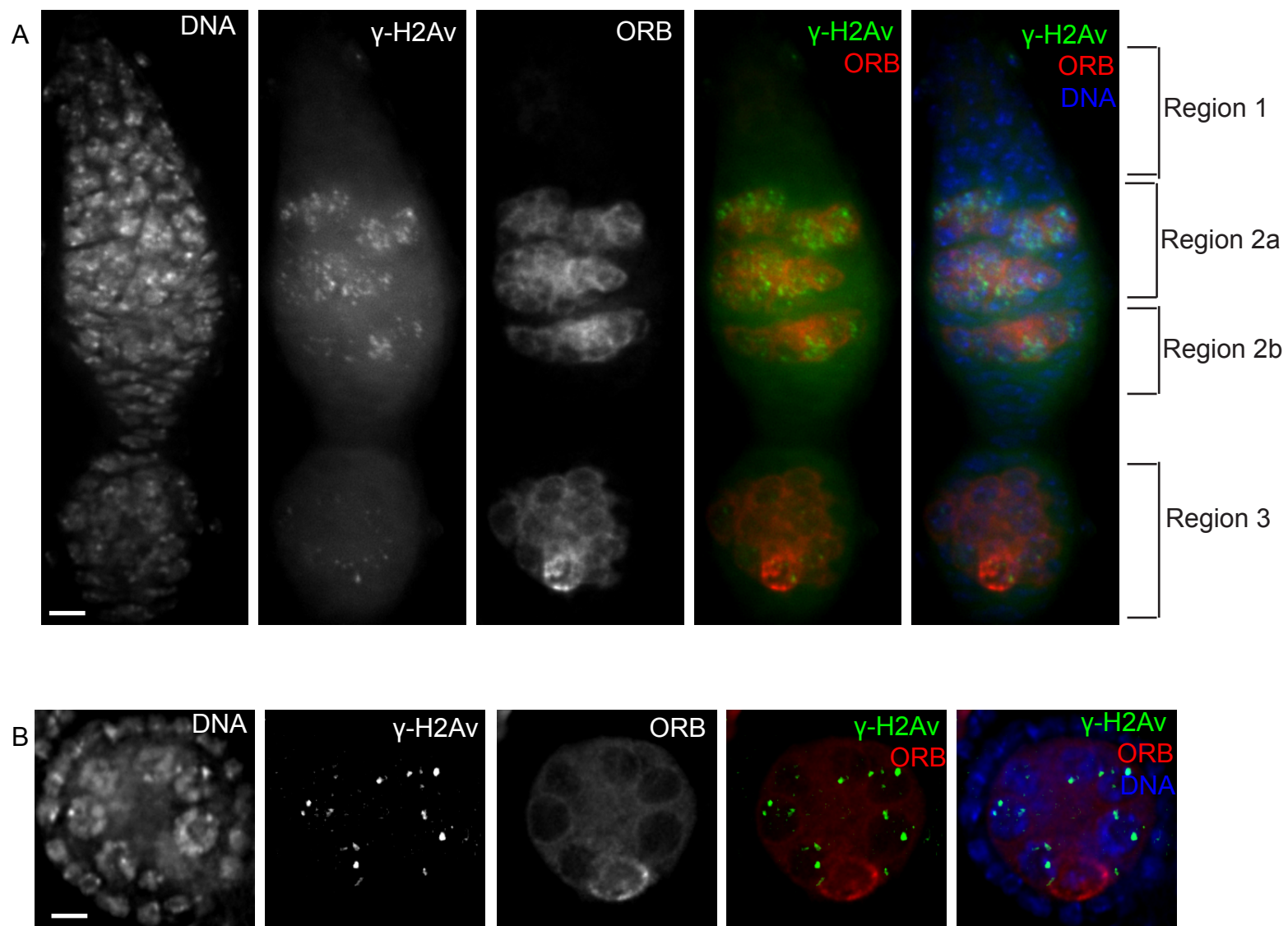

Figure S9

Supplement: Figure S9 — Effect of GFP::VAS expression on γ-H2Av staining in solo pro-oocyte and oocyte nuclei. Female genotype was Df(2L)A267/soloZ2-0198; GFP::VAS/TM6. DSBs were stained by anti- γ-H2Av antibody. Pro-oocytes and oocytes were identified by enriched staining with anti-ORB antibody. DNA was visualized with DAPI. Scale bars: 5 µm. (A) γ-H2Av in germarium. Note the foci in the region 3 oocyte nucleus. (B) γ-H2Av in a stage 2 egg chamber that is from the same ovariole as in (A). Foci are absent in the oocyte although some foci are still present in nearby nurse cells nuclei. (PDF) [file pgen.1003637.s009.pdf]
