# Supplementary material for: The Cohesion Protein SOLO Associates with SMC1 and Is Required for Synapsis, Recombination, Homolog Bias and Cohesion and Pairing of Centromeres in Drosophila Meiosis
Source: PLoS Genet. 2013 Jul 18;9(7):e1003637. doi: 10.1371/journal.pgen.1003637 (PMC3715423; doi:10.1371/journal.pgen.1003637)
Supplement: Figure S2 — Analysis of NDJ and recombination on chromosome 2. (A) solo, cn bw/b vas7 females were used for both the NDJ and recombination crosses. vas7 is a recessive vasa allele [87] that is also a null allele of solo (data not shown). See Table 4 legend for a description of the recombination cross and analysis. To test for chromosome 2 NDJ and to estimate the relative frequencies of sister and homolog NDJ, solo, cn bw/b vas7 females were crossed singly to two C(2)EN, bw sp males. C(2)EN males generate only diplo-2, bw sp and nullo-2 sperm, so viable, euploid progeny are produced only from fertilization by reciprocally aneuploid eggs that result from chromosome 2 NDJ in the female. (B and C) Diplo-2 eggs can carry centromeres either from two homologous or two sister chromatids, so can be used to determine the relative frequencies of homolog and sister NDJ. As shown, these classes can be discriminated by their genotypes with respect to the b and cn markers that flank and are near the centromere. The left panels show the patterns in the absence of crossing over; the right panels show the patterns if there is recombination between the cn and bw loci. Note: the top two genotypes in panel C cannot be distinguished phenotypically but the bottom two genotypes can be. Recombination between b and cn would yield additional genotypes but are not shown because no such events were recovered among the progeny of diplo-2 eggs in either cross. (PDF) [file pgen.1003637.s002.pdf]

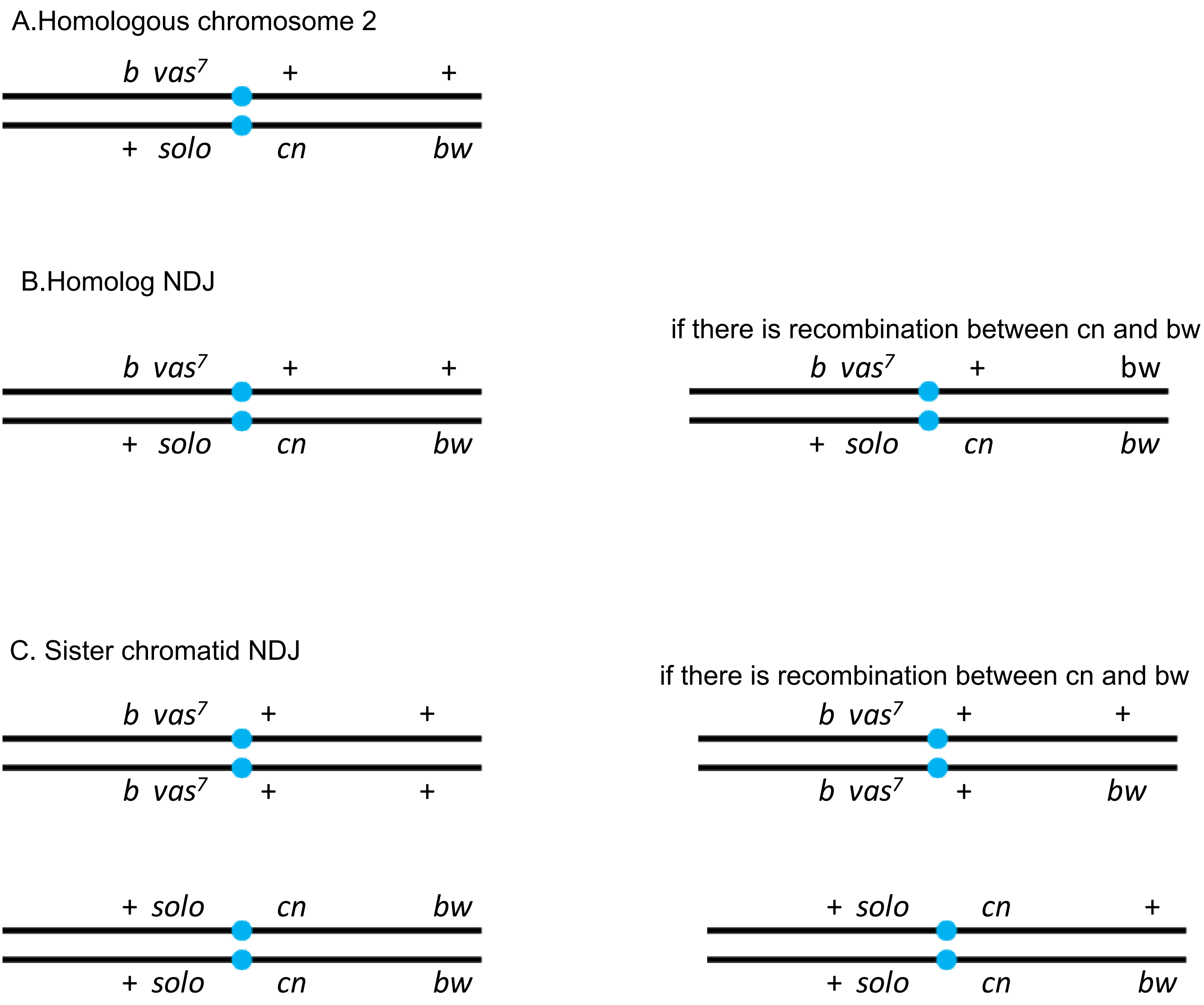
A.Homologous chromosome 2
b vas7 + +
+ solo cn bw
B.Homolog NDJ
b vas7 + +
+ solo cn bw
if there is recombination between cn and bw
b vas7 + bw
+ solo cn bw
C. Sister chromatid NDJ
b vas7 + +
b vas7 + +
+ solo cn bw
+ solo cn bw
if there is recombination between cn and bw
b vas7 + +
b vas7 + bw
+ solo cn +
+ solo cn bw

Figure S2
