# Supplementary material for: The Cohesion Protein SOLO Associates with SMC1 and Is Required for Synapsis, Recombination, Homolog Bias and Cohesion and Pairing of Centromeres in Drosophila Meiosis
Source: PLoS Genet. 2013 Jul 18;9(7):e1003637. doi: 10.1371/journal.pgen.1003637 (PMC3715423; doi:10.1371/journal.pgen.1003637)
Supplement: Table S1 — Results of SOLO transgene NDJ rescue experiments. aThe indicated females were crossed to YSX.YL, In(1)EN, y B males to measure X chromosome NDJ. Df represents Df(2L)A267. nos-GAL4 represents nos-GAL4::VP16. UPS-SOLO represents {UPS-SOLO::Venus}. The numbers in parentheses in lines 6–8 represent the copy number of UPS-SOLO. The generation of {UASp-Venus::SOLO}, {UASp-SOLO::Venus} and {UPS-SOLO::Venus} were described in [53]. b% NDJ = 100×2× (B+ females+y B males)/(N+B+ females+y B males). cN = total number of progeny. Note: the incomplete rescue of X-X NDJ by the UPS-SOLO::Venus construct (lines 6–8) was not due to the C-terminal location of the Venus tag. The same SOLO::Venus protein fully rescued NDJ when expressed under control of the nos-GAL4::VP16 driver (line 3). In tests for rescue of X-Y NDJ in solo males, NDJ was reduced to 5.8%, 3.25% or 1.8% with one, two or three copies, respectively, of {UPS-SOLO::Venus} (unpublished data). (PDF) [file pgen.1003637.s010.pdf]

Table S1. Results of SOLO transgene NDJ rescue experiments.

Genotypes[a]	%NDJ[b]	N[c]
w/+; solo$^{Z2\text{-}0198}$/Df; UASp-Venus::SOLO/nos-GAL4	0%	713
w/+; solo$^{Z2\text{-}0198}$/Df; UASp-Venus::SOLO or *nos-GAL4/TM2*	57.10%	180
w/+; solo$^{Z2\text{-}0198}$/Df; UASp-SOLO::Venus/nos-GAL4	0%	212
w/+; solo$^{Z2\text{-}0198}$/Df; UASp-FH::SOLO/nos-GAL4	0%	1468
w/+; solo$^{Z2\text{-}0198}$/Df; UASp-FH::SOLO or *nos-GAL4/TM2*	51.70%	155
UPS-SOLO; *solo$^{Z2\text{-}0198}$*/Df; *UPS-SOLO* (4)	7.03%	302
UPS-SOLO/w; *solo$^{Z2\text{-}0198}$*/Df; *UPS-SOLO* (3)	8.06%	119
w/w; *solo$^{Z2\text{-}0198}$*/Df; *UPS-SOLO* (2)	10.97%	655
