# Supplementary material for: The Cohesion Protein SOLO Associates with SMC1 and Is Required for Synapsis, Recombination, Homolog Bias and Cohesion and Pairing of Centromeres in Drosophila Meiosis
Source: PLoS Genet. 2013 Jul 18;9(7):e1003637. doi: 10.1371/journal.pgen.1003637 (PMC3715423; doi:10.1371/journal.pgen.1003637)
Supplement: Table S2 — Partial rescue of solo female fertility by GFP::VAS. The indicated females were crossed singly to two y w males to measure fertility and X chromosome NDJ. NDJ was estimated only from the patriclinous sons (derived from nullo-X eggs and X sperm and denoted by “n”) because the matriclinous daughters were indistinguishable from the regular daughters. % NDJ = 100×4n/(N+2n). (PDF) [file pgen.1003637.s011.pdf]

Table S2. Rescue of *solo* female fertility by GFP::VAS.

Genotypes	progeny/female	NDJ	N
w; *solo*$^{Z2\text{-}0198}$*/Df; GFP::VAS/MKRS*	8.8	45.45%	203
w; solo$^{Z2\text{-}0198}$*/Df; GFP::VAS/GFP::VAS*	8.4	47.69%	167
w; solo$^{Z2\text{-}0198}$*/Df; TM6/MKRS*	4.5	48.57%	89
WT	79.1	0%	633
